# Base excision repair AP endonucleases and mismatch repair act together to induce checkpoint-mediated autophagy

**DOI:** 10.1038/ncomms3674

**Published:** 2013-10-24

**Authors:** Tanima SenGupta, Maria Lyngaas Torgersen, Henok Kassahun, Tibor Vellai, Anne Simonsen, Hilde Nilsen

**Affiliations:** 1 The Biotechnology Centre, University of Oslo, PO Box 1125 Blindern, N-0317 Oslo, Norway; 2 Department of Biochemistry, Institute of Basic Medical Sciences, University of Oslo, PO Box 1112 Blindern, N-0317 Oslo, Norway; 3 Department of Genetics, Eötvös Loránd University, Budapest H-1117, Hungary; 4 Present address: Institute of Clinical Medicine, University of Oslo, Oslo, Norway, and Akershus University Hospital, N-1478 Lørenskog, Norway

## Abstract

Cellular responses to DNA damage involve distinct DNA repair pathways, such as mismatch repair (MMR) and base excision repair (BER). Using *Caenorhabditis elegans* as a model system, we present genetic and molecular evidence of a mechanistic link between processing of DNA damage and activation of autophagy. Here we show that the BER AP endonucleases APN-1 and EXO-3 function in the same pathway as MMR, to elicit DNA-directed toxicity in response to 5-fluorouracil, a mainstay of systemic adjuvant treatment of solid cancers. Immunohistochemical analyses suggest that EXO-3 generates the DNA nicks required for MMR activation. Processing of DNA damage via this pathway, in which both BER and MMR enzymes are required, leads to induction of autophagy in *C. elegans* and human cells. Hence, our data show that MMR- and AP endonuclease-dependent processing of 5-fluorouracil-induced DNA damage leads to checkpoint activation and induction of autophagy, whose hyperactivation contributes to cell death.

## Abstract (web summary)

The chemotherapeutic drug 5-fluorouracil causes cell toxicity by inducing DNA lesions. Here, SenGupta *et al.* use *C. elegans* to show that components of the base excision repair and the mismatch repair pathways function together in the response to 5-fluorouracil, resulting in activation of the DNA damage checkpoint and induction of autophagy.

## Graphical abstract

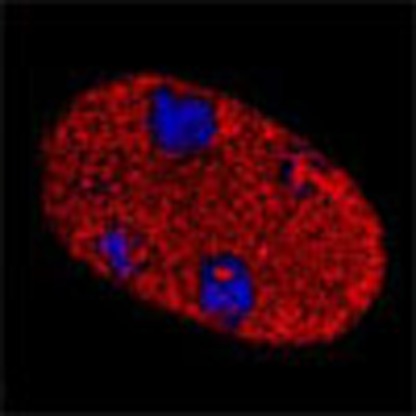

5-Fluorouracil (5-FU) remains a central component of systemic treatment of a wide range of solid cancers in the adjuvant setting^1^. The active metabolite, 5-fluoro-2′-deoxyuridine monophosphate, inhibits thymidylate synthase, which leads to imbalanced nucleotide pools with subsequent incorporation of dUTP and 5-fluoro-2′-dUTP into DNA, and the corresponding ribonucleotides into RNA^2^. As the resulting uracil and fluorouracil (FUra) bases in DNA do not lead to formation of strand breaks directly, it is thought that repair intermediates generated through incomplete or aberrant processing of the original lesions by DNA repair enzymes is the basis for DNA-directed toxicity^1^. Uracil and FUra in DNA are primarily repaired via the base excision repair (BER) pathway^3^. BER is initiated by a uracil–DNA glycosylase (UDG), which excises the damage as a free base. The resulting abasic (apurinic/apyrimidinic (AP)) site is incised by an AP endonuclease to generate a single-strand break, and further processing leads to replacement of one or two nucleotides. All five mammalian UDGs may process uracil or FUra (for review see ref. 4), but conflicting reports exists as to whether they mediate DNA-directed toxicity^2^,^5^,^6^,^7^. Furthermore, no good correlation between BER deficiency and therapeutic response has been observed in clinical material^4^.

In contrast, the DNA mismatch repair (MMR) pathway is an important determinant for 5-FU toxicity, and MMR deficiency is associated with resistance to 5-FU (ref. 1). In the MMR pathway, the MutS complex (MSH-2/MSH-6) binds DNA damage in a mismatch context^8^ and recruits the MutL complex (MLH-1/PMS-2). The MutS/MutL complex then travels away from the mismatch to search for a nick, which is required for loading of an exonuclease (EXO-1) that removes the lesion together with an extended stretch of the surrounding DNA. Thus, processing of DNA damage through the MMR pathway leads to the generation of long stretches of single-stranded DNA, which will be coated by replication protein A (RPA) before replicative polymerases are recruited to fill in the gap^9^.

The mechanistic basis for involvement of MMR in response to 5-FU is puzzling, as 5-FU primarily leads to incorporation of uracil and FUra opposite adenine to generate BER substrates. The high degree of redundancy among UDGs effectively prevents further clarification of the division of labour between BER and MMR in activation of DNA-directed toxicity in human cells. Thus, we used *Caenorhabditis elegans* as a model to investigate the function of the two DNA repair pathways in eliciting 5-FU toxicity. *C. elegans* has only one characterized UDG, the enzyme UNG-1 (ref. 10), and has significantly contributed to our understanding of the role of DNA repair pathways in initiating DNA damage response (DDR) signalling in response to misincorporated uracil^11^. Furthermore, as *C. elegans* also has only one MutS complex, it allows genetic interrogation of the separate role of BER and MMR in eliciting DNA-mediated toxicity in response to 5-FU.

Here we show that the DNA damage recognition complex of MMR, but not BER, acts as a sensor of DNA damage induced by 5-FU. Furthermore, epistasis analyses show that the BER AP endonucleases APN-1 and EXO-3 function in the same pathway as MMR to induce toxicity in response to 5-FU. Immunohistochemical analyses suggest that EXO-3 generates DNA nicks required for MMR activation, whereas APN-1 is required for checkpoint activation. Processing of DNA damage via this pathway, in which both BER and MMR enzymes are required, leads to induction of autophagy in *C. elegans* and in human U2OS cells. This suggests that failure to process 5-FU-induced DNA damage via this pathway is a basis for resistance.

## Results

### *C. elegans* MMR and BER mutants are resistant to 5-FU

**Figure 1:**
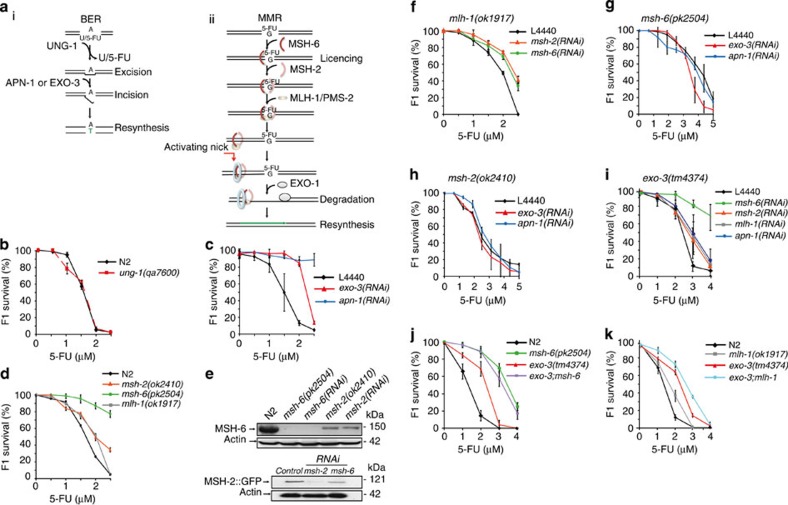
Epistatic interactions between MutS and the BER AP endonucleases with respect to 5-FU sensitivity. (**a**) Cartoons showing schemes of the (i) BER and (ii) MMR pathways. F1 survival (**b**) in N2 wild type (black diamonds) and *ung-1* mutants (red squares), (**c**) following the depletion of EXO-3 (red triangles), APN-1 (blue circles) or control RNAi (L4440, black diamonds) in the N2 wild-type background, and (**d**) in *msh-2(ok2410)* (orange triangles), *msh-6(pk2504)* (green circles) and *mlh-1(ok1917)* (grey squares) mutants. (**e**) Western blottings showing expression of MSH-6 protein in the wild type strain grown on *E. coli* expressing control (N2) or the indicated RNAi, as well as in *msh-6(pk2504)* and *msh-2(ok2410)* mutants (top panel), and expression of MSH-2::GFP fusion protein in transgenic worms grown on *E. coli* expressing RNAi for MSH-2, MSH-6 or empty vector control as indicated (lower panel). Actin was used as the loading control. (**f**) F1 survival after depletion of MSH-2 and MSH-6 in the *mlh-1(ok1917)* mutant. (**g**,**h**) F1 survival after depletion of the AP endonucleases EXO-3 and APN-1 in (**g**) the *msh-6(pk2504)* or (**h**) *msh-2(ok2410)* mutant background. Kruskal–Wallis one-way analysis on ranks (*P*=0.963 and *P*=0.985 in **h** and **g**, respectively). Mann–Whitney rank-sum test failed to identify a difference in median survival after depleting EXO-3 in the *msh-6* mutant (**g**, *P*=0.929). (**i**) F1 survival following depletion of the indicated genes in *exo-3(tm4374)* mutants. (**j**) F1 survival in *msh-6(pk2504)*, *exo-3(tm4374)* and *exo-3;msh-6* double mutants. (**k**) F1 survival in *exo-3(tm4374)*, *mlh-1(ok1917)* and *exo-3;mlh-1* double mutants. All survival curves (**b**–**d**,**f**–**k**) show the mean±s.d. for each data point from three independent experiments.

Removal of uracil or FUra bases from DNA is generally believed to be required for generating the repair intermediates leading to DNA-directed toxicity induced by 5-FU. Thus, we asked whether mutants of the lesion recognition enzymes in the BER or MMR (Fig. 1a) pathways led to 5-FU resistance in *C. elegans.*

A null mutant of the UNG-1 enzyme, *ung-1(qa7600*; ref. 11, also see Supplementary Fig. S1 for description of the mutant alleles) was as sensitive to 5-FU as the wild type (N2; Fig. 1b). In contrast, pronounced 5-FU resistance was observed after RNA interference (RNAi)-mediated depletion of the AP endonucleases EXO-3 and APN-1 (Fig. 1c). As the AP endonucleases act downstream of UNG-1 in the BER pathway (Fig. 1a), this surprising result indicates that DNA-mediated toxicity proceeds via an UNG-1-independent alternative pathway that also requires AP endonucleases.

An MSH-6 mutant, *msh-6(pk2504)* was remarkably resistant to 5-FU (Fig. 1d). After treating nematodes with 2 μM 5-FU, >80% of the *msh-6(pk2504)* mutant offspring developed into adults compared with <5% of the wild type (Fig. 1d). In contrast, only 50% of MSH-2 mutants, *msh-2*(*ok2410*), developed into adults (Fig. 1d) and *mlh-1(ok1917)* mutant animals were as sensitive as *msh-2(ok2410)* mutants (Fig. 1d). This surprising intermediate phenotype of *msh-2(ok2410)* mutants would be consistent with aberrant processing of 5-FU-induced DNA damage initiated by MSH-6 without appropriate recruitment of downstream repair factors. This was unexpected given that MSH-2 acts together with MSH-6 in the MutS complex and the stability of human MSH-6 depends on MSH-2 (ref. 12). However, western blot analysis confirmed that in *C. elegans*, MSH-6 was expressed in *msh-2(ok2410)* mutants and in the wild type after RNAi-mediated depletion of MSH-2, but was not detected in *msh-6(pk2504)* mutants and *msh-6(RNAi*) animals (Fig. 1e). Moreover, expression of an MSH-2::GFP translational fusion protein could be detected after depleting MSH-6 but not MSH-2 (Fig. 1e). As depletion of MSH-6 or MSH-2 was epistatic to the *mlh-1(ok1917)* mutation (Fig. 1f), we conclude that processing of 5-FU-induced DNA damage through the MMR pathway is the basis for DNA-directed toxicity, and that MSH-6 seems to have an upstream function in lesion processing (see Supplementary Fig. S2 for RNAi efficiency and confirmation that phenotypes induced by RNAi resemble mutant phenotypes).

To test whether the AP endonucleases act in the same pathway as MMR to mediate 5-FU-directed toxicity, we depleted APN-1 and EXO-3 in *msh-6* and *msh-2* mutant animals (Fig. 1g,h, respectively). In both cases epistasis was observed, as no statistically significant difference in median survival was found between the groups. Thus, the BER AP endonucleases act in the same pathway as MMR to mediate 5-FU toxicity. Further epistasis analyses in *exo-3(tm4374)* mutants suggested that MSH-6 operates upstream of EXO-3, whereas MSH-2, MLH-1 and APN-1 probably act downstream of EXO-3, as their depletion resulted in 5-FU sensitivity indistinguishable from the *exo-3(tm4374)* mutant-fed control RNAi (Fig. 1i). Analyses in *exo-3;msh-6* and *exo-3;mlh-1* double mutants (Fig. 1j,k, respectively) confirmed the epistasis between EXO-3, and both MSH-6 and MLH-1. Finally, the *msh-6* single and *exo-3;msh-6* double mutants showed most pronounced tolerance to 5-FU, supporting an upstream role of MSH-6 in eliciting 5-FU toxicity. Hence, the epistasis experiments support a model where the BER AP endonucleases function downstream of MutS to elicit DNA-directed toxicity in response to 5-FU.

### DNA repair-dependent checkpoint activation

Next, we asked whether the processing of 5-FU-induced DNA damage led to apoptosis. Despite the appearance of cytological markers for DDR activation (Supplementary Fig. S3a,b), apoptotic cell death was not observed upon 5-FU treatment neither in the germline (Supplementary Fig. S3c)^13^ nor in embryos (Supplementary Fig. S3d).

**Figure 2:**
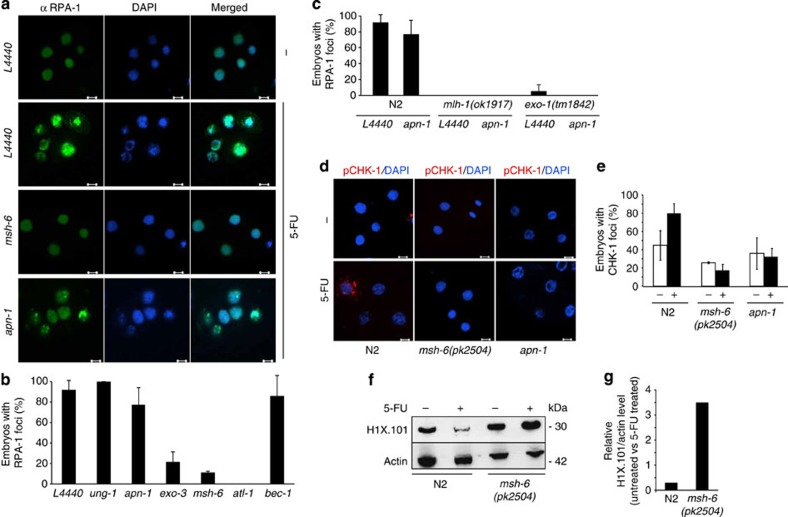
DNA damage checkpoint activation in *C. elegans.* (**a**) Immunofluorescence images showing anti-RPA-1-positive foci in response to 5-FU after depleting the indicated genes by RNAi or empty vector control RNAi (L4440; scale bars, 5 μm). (**b**) Quantification of the fraction (%) of 5-FU-treated embryos that contain RPA-1-positive foci. (**c**) Quantification of the fraction (%) of 5-FU-treated embryos that contain RPA-1-positive foci in N2, *mlh-1(ok1917)* and *exo-1(tm1842)* mutants fed control (L4440) or RNAi targeting APN-1 (*apn-1*). (**d**) Immunofluorescence showing CHK-1 phosphorylation at Ser139 in response to 5-FU in N2 but not in *msh-6(pk2504)* mutants or in N2 after *apn-1(RNAi)* (scale bars, 5 μm). (**e**) Quantification of the fraction (%) of 5-FU-treated embryos with phospho-CHK-1 foci. (**f**) Western blottings showing H1X.101 levels in N2 and *msh-6* mutants, treated (+) or not (−) with 5-FU. Actin was used as loading control. (**g**) Quantification of H1X.101 levels relative to actin in control versus 5-FU-treated worms. (**b**,**c**,**e**,**g**) Bar graphs show the mean±s.d. from three independent experiments.

However, RPA-1-positive foci, which are early markers for DDR activation, accumulated in 5-FU-treated embryos (Fig. 2a). RPA-1 foci were observed in animals depleted for UNG-1 (Fig. 2b). In contrast, RPA-1 focus formation depended on MSH-6 (Fig. 2a,b and Supplementary Table S1) and, on further processing, through the MMR pathway, as RPA-1 foci did not form in the *mlh-1(ok1917)* mutant and were suppressed in the *exo-1(tm1842)* mutant (Fig. 2c and Supplementary Table S2). Hence, we conclude that RPA-1 focus formation depends on processing 5-FU-induced DNA damage through the MMR pathway.

The epistasis of MSH-6 between EXO-3 and APN-1 (Fig. 1g,j) could be explained if these endonucleases provide the DNA nicks required for the loading of EXO-1. If so, we would expect that depletion of the BER AP endonucleases prevented RPA-1 focus formation. This was indeed the case for EXO-3 (Fig. 2b and Supplementary Table S1). In contrast, depletion of APN-1, which, according to our epistasis analysis, acts downstream of EXO-3, did not significantly suppress RPA-1 focus formation in the wild-type background (Fig. 2a,b). However, depletion of APN-1 in *exo-1(tm1842)* mutants completely abrogated RPA-1 focus formation (Fig. 2c and Supplementary Table S2). The different outcomes with respect to RPA-1 focus formation confirmed that APN-1 and EXO-3 have non-redundant functions. The finding that EXO-3 was required for RPA-1 filament formation suggested that it might contribute to generate nicks required for the activation of MMR.

RPA-1 filament formation in response to DNA damage is an indication of DNA damage checkpoint activation that, in 5-FU-treated human cells, involves CHK-1 phosphorylation^14^. 5-FU induced-phosphorylation of CHK-1 in wild-type *C. elegans* but not in *msh-6(pk2504)* mutant embryos (Fig. 2d,e). Interestingly, CHK-1 phosphorylation was not induced in APN-1-depleted embryos (Fig. 2d,e) despite the presence of RPA-1 foci. The immunohistochemical analysis therefore supports that checkpoint activation depends on DNA repair. Although MSH-6 function is required both for RPA-1 focus formation and CHK-1 phosphorylation, APN-1 is dispensable for RPA-1 filament formation.

The absence of RAD-51-positive foci (Supplementary Table S3) showed that checkpoint activation was not likely to be a result of DNA double-stranded break (DSB) formation. However, compared with untreated nuclei, 5-FU-treated nuclei were larger with faint DAPI (4′,6-diamidino-2-phenylindole) staining already at the four- to six-cell stage embryo (Fig. 2a). Taken together, this suggested that 5-FU primarily leads to chromatin decompaction rather than DSB formation. Consistently, the maximum nuclear diameter, defined by the perimeter of lamin-1 (LMN-1) staining in the four-cell stage embryo, increased from 8.2 to 9.0 μm (Student’s *t*-test, *P*<0.05) by 5-FU treatment concomitant with reduced DAPI staining intensity in an MSH-6-dependent manner (Supplementary Fig. S4). Chromatin decompaction was confirmed by the loss of linker histone H1X in 5-FU-treated embryos (Fig. 2f,g). H1X levels were not reduced upon 5-FU treatment in the *msh-6(pk2504)* mutant. This gives independent confirmation that chromatin decompaction and checkpoint activation depended on DNA-repair-mediated processing of 5-FU-induced DNA damage.

### 5-FU induces autophagy in *C. elegans*

**Figure 3:**
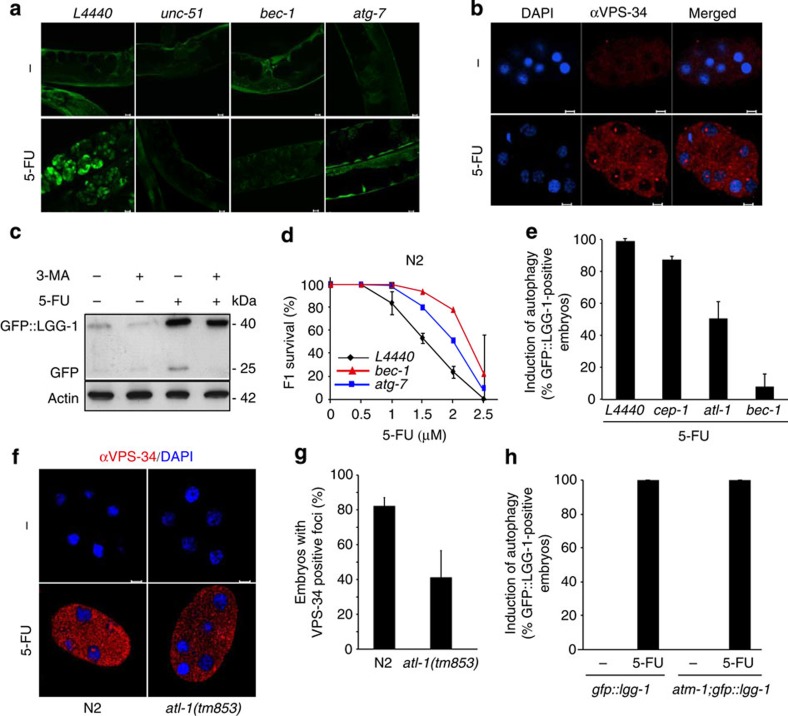
Activation of autophagy in 5-FU-treated *C. elegans.* (**a**) Autophagy induction in response to 5-FU measured in a GFP::LGG-1 reporter strain fed control RNAi (L4440) or RNAi targeting the indicated genes (scale bars, 10 μm). (**b**) Immunofluorescence showing anti-VPS-34 staining in dissected embryos in the absence or presence of 5-FU (scale bar, 5 μm). (**c**) Western blot analysis of embryonic extracts with and without the addition of 5-FU or the autophagy inhibitor 3-MA, detecting the ~40-kDa GFP::LGG-1 fusion protein and the cleaved ~25-kDa product. (**d**) F1 survival measured after depletion of BEC-1 (red triangles) and ATG-7 (blue squares) as compared with worms fed control RNAi (black diamonds). The survival curve shows the mean±s.d. for each data point from three independent experiments. (**e**) Induction of autophagy (% GFP-positive embryos) following depletion of the indicated genes. (**f**) Immunofluorescence showing anti-VPS-34 staining in N2 and *atl-1(tm853)* embryos in the absence or presence of 5-FU (scale bar, 5 μm). (**g**) The fraction (as % of untreated control) of embryos with VPS-34-positive foci following 5-FU treatment. (**h**) Induction of autophagy (% GFP-positive embryos) in control (GFP::LGG-1) and *atm-1(gk186)* mutants (*atm-1;* GFP::LGG-1). (**e**,**g**,**h**) Bar graphs represent mean±s.d. from three independent experiments.

As we recently showed that uracil incorporation induces cell death with both apoptotic and autophagic features^15^, we tested whether 5-FU may induce autophagy as an alternative cell death response^16^. During the process of macroautophagy (hereafter, it is referred to as autophagy), parts of the cytoplasm are sequestered by a double lipid layer that grows to form a small vesicle (autophagosome). The autophagosome then fuses with a lysosome to generate an autolysosome, in which the cargo is degraded by acidic hydrolases. To monitor the activation of autophagy, we used the transgenic reporter strain expressing LGG-1 (*C. elegans* Atg8/LC3) in fusion with green fluorescent protein (GFP). LGG-1 remains associated with autophagic membranes from the early nucleation step until fusion with lysosomes. Increased LGG-1 expression and appearance of GFP::LGG-1-positive puncta indicated that autophagy was activated in the embryos of 5-FU-treated hermaphrodites (Fig. 3a). LGG-1 accumulation was dependent on the core autophagic machinery, as it was suppressed after depletion of UNC-51 (*C. elegans* ULK1/Atg1 homologue), a serine/threonine kinase that regulates autophagy induction; the class III phosphatidylinositol 3-kinase (PI3K) complex subunit BEC-1 (*C. elegans* beclin1/Atg6), which is required for vesicle nucleation; and ATG-7, an E1-like enzyme involved in conjugation of the ubiquitin-like proteins LGG-1 and ATG-12 to autophagic membranes (Fig. 3a). Depletion of LGG-1 itself suppressed puncta formation in a BEC-1::GFP reporter strain (Supplementary Fig. S5a–c). To exclude the possibility that 5-FU-induced autophagy could be an artefact of the transgenic reporter system used, we monitored the expression of the endogenous VPS-34 protein (a homologue of human class III PI3K) that interacts with BEC-1 (17). Confirming autophagy activation in response to 5-FU treatment, an overall increase in VPS-34 expression was observed together with the appearance of VPS-34-positive puncta around the nuclear periphery and in the cytoplasm (Fig. 3b). Distinct VPS-34 foci were also observed in the nucleoli, indicating that VPS-34 shuttles between the nucleus and cytoplasm, similar to what was previously described for its interaction partner Beclin 1 (ref. 18). Furthermore, as LGG-1 itself is degraded as part of the autophagic process, the appearance of a 25-kDa band corresponding to GFP in 5-FU-treated embryos, which disappeared upon addition of the PI3K inhibitor 3-methyladenine (3-MA), confirmed increased autophagic flux upon 5-FU treatment (Fig. 3c). Although autophagy primarily acts as a prosurvival mechanism following various forms of stress, including starvation, excessive activation of autophagy can promote cell death^15^,^16^. The significant increase in progeny survival in animals depleted for *bec-1* and *atg-7* before 5-FU treatment showed that autophagy, in this case, contributes to toxicity (Fig. 3d).

A connection was recently described between the ATR (Ataxia telangiectasia and Rad3 related) DNA damage checkpoint and activation of autophagy in yeast^19^. Depletion of ATL-1 (the *C. elegans* ATR orthologue^20^) reduced autophagy induction by about 50% in *C. elegans* (Fig. 3e and Supplementary Table S4), whereas no effect was seen upon loss of CEP-1 (the *C. elegans* p53 orthologue; Fig. 3e and Supplementary Table S4). In *atl-1(tm853)* mutants, there was weaker cytoplasmic anti-VPS-34 staining and the fraction of embryos with VPS-34-positive puncta was halved compared with the N2 control (Fig. 3f,g), supporting a partial requirement of ATL-1 for 5-FU-induced autophagy. In contrast, the fraction of embryos with GFP::LGG-1-positive puncta was indistinguishable in the *atm-1(gk186)* mutant background and the wild-type background. (Fig. 3h).

### DNA repair-dependent induction of autophagy

**Figure 4:**
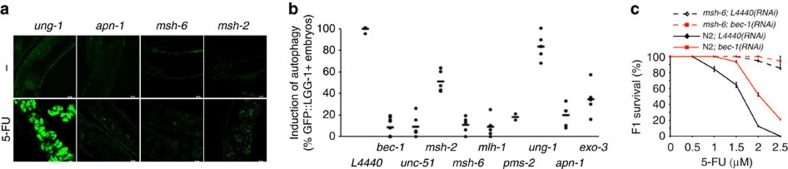
DNA repair-dependent induction of autophagy in response to 5-FU. (**a**) Induction of autophagy in response to 5-FU in the GFP::LGG-1 reporter strain fed on RNAi targeting the indicated DNA repair genes (scale bars, 10 μm). (**b**) The fraction (as % of untreated control) of hermaphrodites having embryos with excessive GFP expression following 5-FU treatment. Autophagy induction in independent experiments (circles) and the mean (line) are presented. (**c**) F1 survival after control (black diamonds) or *bec-1* RNAi (red squares) in N2 and *msh-6(pk2504)* mutants. The survival curve shows the mean±s.d. for each data point from three independent experiments.

Next, we asked whether autophagy induction, similar to chromatin decompaction, RPA-1 focus formation, and CHK-1 phosphorylation also depended on DNA repair. This was addressed by counting embryos with GFP::LGG-1-positive foci in animals after RNAi-mediated depletion of individual DNA-repair proteins. All embryos in 5-FU-treated control animals were GFP-positive and depletion of UNG-1 had a minor effect (Fig. 4a,b and Supplementary Table S4). Confirming the survival experiments (Fig. 1c,d), 51% of MSH-2-depleted embryos were GFP positive, whereas depletion of MSH-6, MLH-1, PMS-2, APN-1 and EXO-3 dramatically reduced the number of GFP-positive embryos (Fig. 4b and Supplementary Table S4). Similar results were obtained using accumulation of VPS-34 as a read-out for autophagy activation (Supplementary Fig. S5d,e). Thus, 5-FU-induced autophagy depends on DNA repair. As predicted, as depletion of core autophagy genes attenuated 5-FU toxicity (Fig. 3d), suppression of autophagy further improved survival in the already-tolerant *msh-6(pk2504)* mutant (Fig. 4c).

**Figure 5:**
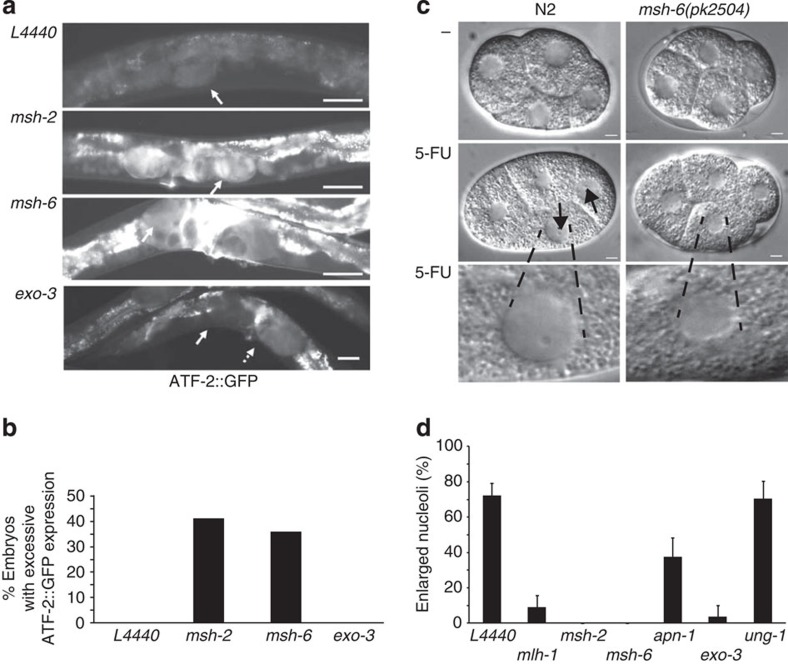
Loss of MutS induces ATF-2 expression and attenuates nucleolar stress phenotypes. (**a**) Expression of ATF-2::GFP in 5-FU-treated hermaphrodites fed control or RNAi targeting the indicated genes (scale bars, 20 μm). (**b**) The number of animals harbouring embryos with excessive ATF-2::GFP expression was scored in at least 150 animals and given as the fraction (%) of the total. (**c**) N2 or *msh-6(pk2504)* embryos were continuously monitored under differential interference contrast microscopy (scale bars, 5 μm) from the three-cell stage until completion of the four-cell stage. Magnified images of representative nuclei are shown. (**d**) The fraction of embryos with visible, enlarged nucleoli (arrows) after 5-FU treatment was scored. Bar graphs represent mean±s.d. from three independent experiments.

We also monitored the expression of the ATF-2 transcription factor, which negatively regulates the expression of autophagy genes such as *lgg-1* and *bec-1* in *C. elegans*^15^. In line with an upstream function of the MutS complex, we found that depletion of *msh-2* and *msh-6* resulted in 41% and 36% (93 out of 225, or 54 out of 150 embryos scored) of embryos showing excessive ATF-2 expression, which was not seen in the control (1,825 embryos scored; Fig. 5a,b). In contrast, no excessive ATF-2::GFP expression was observed in *exo-3* or *apn-1*-depleted worms (150 embryos scored). Hence, the MutS complex functions to instigate the cellular response cascade leading to DNA-mediated toxicity in response to 5-FU.

### DNA repair-dependent ribosome biogenesis defect

The DNA repair-dependent accumulation of VPS-34 in the nucleoli indicated that processing of 5-FU-induced DNA damage led to specific problems in this compartment. It is known that 5-FU inhibits ribosome biogenesis at the level of late ribosomal RNA processing^21^. These phenotypes are commonly interpreted to be resulting from incorporation of 5-fluorouridine triphosphate into RNA^2^. As regulation of ribosome biogenesis is tightly linked to regulation of chromatin condensation state, we suspected that MMR-dependent chromatin decompaction in response to 5-FU might contribute to these phenotypes. In *C. elegans*, ribosome biogenesis defects can be monitored as the appearance of visible (enlarged) nucleoli during early embryonic development. The appearance of enlarged nucleoli in ~70% of the four-cell stage embryos treated with 5-FU (Fig. 5c) is therefore a phenotype consistent with an RNA biogenesis defect. The enlarged nucleoli phenotype is in this case, however, unlikely to be a consequence of incorporation of 5-fluorouridine triphosphate into RNA, as it was absent in the *msh-6* mutant (Fig. 5c,d). Moreover, the enlarged nucleoli phenotype was not observed after depletion of MSH-2, MLH-1 and EXO-3, and was partially suppressed by APN-1 depletion. In contrast, enlarged nucleoli were seen at wild-type levels after depletion of UNG-1 (Fig. 5d). Hence, the appearance of enlarged nucleoli depended on DNA repair, and its suppression coincided with the suppression of nucleolar VPS-34 staining and failure to induce autophagy. This suggests that RNA- and DNA-mediated 5-FU toxicity have a common mechanistic component.

### 5-FU induces DNA repair-dependent autophagy in human cells

**Figure 6:**
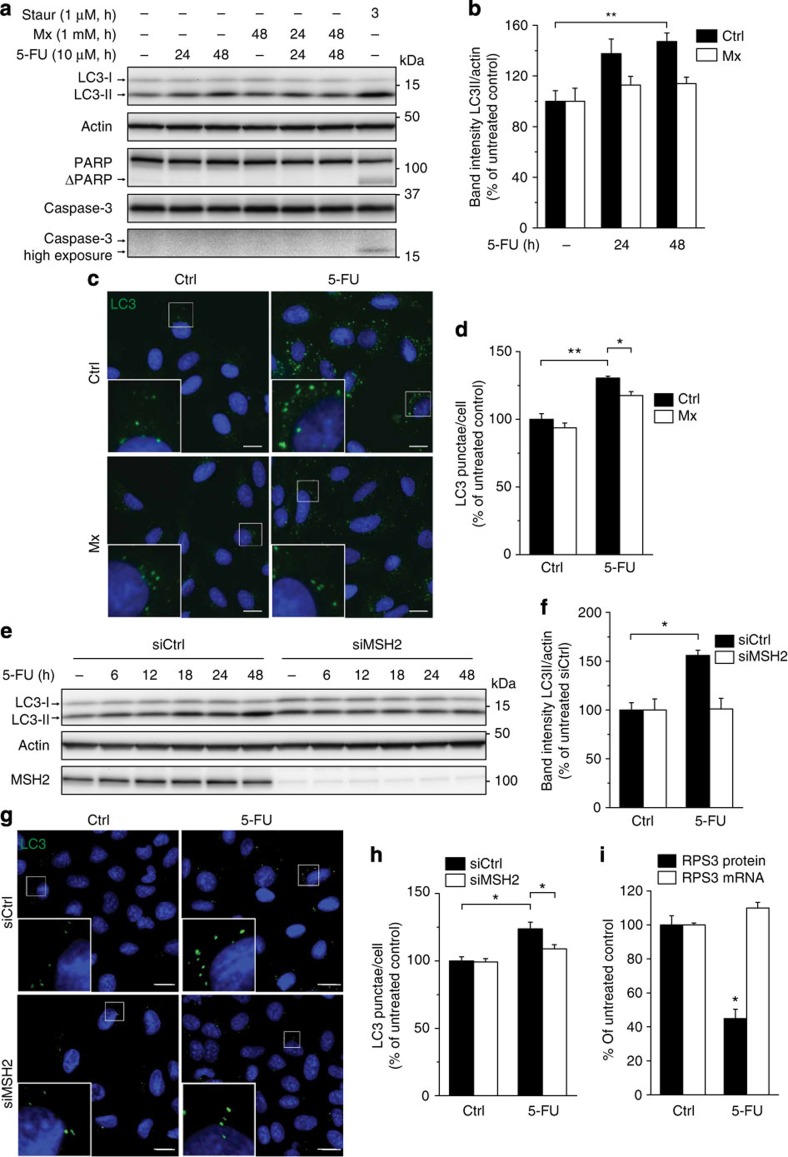
DNA repair-dependent autophagy induction upon 5-FU treatment in human cells. (**a**) Western blots showing autophagy induction by monitoring LC3 levels in whole-cell extracts from U2OS cells treated or not with 5-FU alone or in combination with Mx for the indicated time. Staurosporin was used as a positive control for apoptosis induction shown by the appearance of cleaved fragments of caspase-3 (17 and 19 kDa) and poly (ADP-ribose) polymerase 1 (PARP1; ΔPARP, 85 kDa). α−Actin was used as loading control. (**b**) Quantification of LC3-II levels in cells treated as in **a**. Intensities of the lower LC3 band (LC3-II) was normalized to those of actin. (**c**) Autophagy induction demonstrated by accumulation of LC3-positive puncta in U2OS cells treated or not with 5-FU alone or in combination with Mx for 48 h. The cells were fixed and prepared for immunofluorescence of LC3 (green). DAPI staining is shown in blue. Scale bar, 20 μm. (**d**) Quantification of LC3-positive puncta in cells treated as in **c**. In each separate experiment, 200–1,600 cells were scored per condition. (**e**) Western blots showing absence of LC3-II accumulation in U2OS cells transfected with siRNA against MSH-2. The same blot probed with anti-MSH-2 antibodies confirm efficient knockdown. (**f**) Quantification of LC3-II levels in cells transfected with control or MSH-2 siRNA with or without FU treatment for 24 h. (**g**) Immunofluorescence showing reduced accumulation of LC3 puncta in U2OS cells transfected with siRNA against MSH-2. (**h**) Quantification of LC3-positive puncta in cells treated as in **g**. (**i**) Quantification of normalized RPS3 protein level (black bars) or RPS3 mRNA level (white bars) in U2OS cells after 48 h 5-FU treatment. RPS3 mRNA levels were determined by quantitative real-time reverse transcriptase–PCR. Graphs (**b**,**d**,**f**,**h**,**i**) show mean values±s.e.m. quantified from at least three independent experiments. Student’s *t*-tests were performed to assess the significance between treatment groups in all panels as indicated; *P<0.05; **P<0.01.

To confirm the relevance of our finding for human cells, we monitored induction of autophagy in human U2OS cells treated with 10 μM 5-FU. Upon induction of autophagy, cytosolic LC3 (orthologous to *C. elegans* LGG-1; LC3-I, 18 kDa band) is cleaved and bound to phosphatidylethanolamine in the autophagic membranes (LC3-II, 16 kDa band) and, as LC3-II remains bound to autophagic membranes throughout the pathway, it can be used as a marker for autophagy. A weak but consistent induction of autophagy was seen following 18 h of exposure to 5-FU, with maximum LC3-II expression between 24 and 48 h (Fig. 6a,e). Autophagy was not activated secondarily to apoptosis as neither poly (ADP-ribose) polymerase 1 nor caspase-3 cleavage was observed. Induction of autophagy was suppressed by inhibiting the major mammalian AP endonuclease APE1 by methoxyamine (Mx; Fig. 6a,b). Similarly, a reduction of 5-FU-induced LC3-positive puncta was seen in the presence of Mx in U2OS cells (Fig. 6c,d). Induction of autophagy by 5-FU also depended on MMR in human cells, as depletion of MSH-2 effectively suppressed LC3-II induction (Fig. 6e,f) and formation of LC3-positive puncta (Fig. 6g,h). Similar results were obtained after depletion of MSH-6 by short interfering RNA (siRNA) as expected, as the stability of MSH-2 protein depends on MSH-6 in mammalian cells^12^ (Supplementary Fig. S6a).

Finally, we asked whether nucleolar proteins or nucleic acids were degraded by autophagy as suggested by the nucleolar VPS-34-positive foci seen in 5-FU-treated *C. elegans*. Expression of the RPS3 subunit of the 40S small ribosomal particle, which shuttles between the nucleolus and cytoplasm^22^, was gradually lost in 5-FU-treated U2OS cells (Fig. 6i and Supplementary Fig. S6b), although RPS3 mRNA levels were unaffected (Fig. 6i). Indeed, depletion of Ulk1, a kinase required for induction of autophagy, prevented the 5-FU-induced degradation of RPS3 (Supplementary Fig. S6c,d) confirming that autophagic activity contributed to RPS3 degradation.

In summary, we have identified a novel conserved mechanism for 5-FU-induced toxicity. MutS and the BER AP endonucleases are required to elicit DNA-directed toxicity, leading to checkpoint activation and autophagy induction in response to 5-FU in *C. elegans* embryos and in human cell lines. Epistasis analyses in *C. elegans* are consistent with MutS and the BER AP endonucleases acting in the same pathway with EXO-3, providing a nick for activation of the MMR pathway *in vivo*.

## Discussion

Preclinical and clinical evidence show that MMR deficiency is associated with poor therapeutic response to a wide range of cytotoxic drugs^23^. It is not clear whether this effect is due to a classical DNA repair function of MMR or a role for MMR in signalling DNA damage^4^. The DNA damages induced by 5-FU can be repaired by BER and MMR, but little is known about the nature of the crosstalk between these DNA repair pathways beyond the fact that they share substrates. Here we present genetic and molecular evidence to show that the MMR MutS complex is the upstream requirement for DNA-mediated toxicity. Importantly, we show that the BER AP endonucleases EXO-3 and APN-1 act in the same pathway as classical MMR in eliciting DNA-directed toxicity in response to 5-FU in *C. elegans* and in a human cell line. We present evidence that EXO-3, the *C. elegans* orthologue of human APN-1, generates the nicks required for MMR activation *in vivo*. Processing of 5-FU-induced DNA damage via this pathway led to autophagy activation, which coincided CHK-1 phosphorylation, showing that autophagy is activated as a consequence of DNA damage processing and checkpoint activation. Failure to induce autophagy correlated with resistance to 5-FU. Autophagy was, as such, a determinant for sensitivity.

We identified a specific requirement for MMR and BER to elicit 5-FU toxicity. Depletion of genes participating in other DNA repair pathways did not result in similar resistance (Supplementary Fig. S7). Genetic studies in *Saccharomyces cerevisiae* suggest that the nucleotide excision repair (NER) pathway may contribute to repair of AP sites in the absence of BER^24^, which might explain the somewhat increased 5-FU sensitivity of a mutant in XPA-1, a DNA damage binding protein required for NER (Supplementary Fig. S7a). In contrast, depletion of ERCC-1 or XPF-1, two proteins that function together as a structure-specific endonuclease in the NER pathway, resulted in a mild tolerance (Supplementary Fig. S7b), but this effect might be due to the role of ERCC-1/XPF-1 in repair of DSB^25^ rather than in NER. Similar tolerance was also observed after depleting proteins acting in the two main DSB repair pathways, homologous recombination and non-homologous end joining (Supplementary Fig. S7c,d, respectively). These results suggest that both NER and DSB repair pathways may process DNA-repair intermediates generated by MMR and BER in *C. elegans*, but that they have modest effects on overall toxicity. Moreover, our results revealed that the MutS complex, in particular the MSH-6 protein, acts as the initial sensor of 5-FU-induced DNA damage, as none of the cellular effects induced by 5-FU in the wild-type background was seen in MSH-6-deficient animals; *msh-6* mutants showed no chromatin decompaction, failed to activate the DNA damage checkpoint as measured by RPA-1 focus formation and CHK-1 phosphorylation, and failed to induce autophagy. The nature of the *in vivo* substrates for cytotoxic MMR processing remains to be demonstrated. However, as only BER is expected to be able to process 5-FUra/Ura base paired with adenine, these lesions probably do not contribute much to DNA-mediated toxicity, as lack of UNG-1 does not affect overall survival. Nevertheless, UNG-1-initiated BER probably repairs 5-FU-induced DNA damage, as depletion of *exo-3* and *apn-1* gave less-effective suppression of toxicity in *ung-1* mutants than in the wild type (Supplementary Fig. S2b), and depletion of UNG-1 improved survival in the *exo-3* mutant (Supplementary Fig. S2d). Interestingly, the sharp reduction in F1 survival in *exo-3*-depleted animals treated with higher concentrations of 5-FU (Fig. 1c) was lost in *ung-1* mutants (Supplementary Fig. S2b) and, conversely, upon depletion of UNG-1 in *exo-3* mutants (Supplementary Fig. S2d). This suggests that BER becomes more important at higher 5-FU concentrations as was previously observed in human cell lines^26^. BER and MMR both remove 5-FUra/Ura in a mismatched context. Some competition for substrates is supported experimentally with depletion of MSH-2 and MSH-6 being epistatic in *ung-1* mutants (Supplementary Fig. S2c). However, MMR is the only pathway expected to be able to detect the mismatched normal bases expected to arise from the nucleotide pool imbalance induced by 5-FU. Hence, MMR might respond to many different substrates in 5-FU-treated cells and it is likely to be that all of these substrates contribute to DNA-mediated toxicity. Importantly, only MMR-initiated processing was associated with checkpoint activation and downstream DNA damage signalling. This is consistent with resistance to 5-FU-based chemotherapy in MMR-defective human cells and tumours^4^, whereas loss of UNG-1 and SMUG1 DNA glycosylases have little effect^2^,^5^. The requirement of mammalian TDG (thymine–DNA glycosylase) for DNA-mediated toxicity^7^ might reflect that TDG might compete with MMR for mismatched substrates^27^.

It was recently demonstrated that MMR may act outside of S-phase in response to cytotoxic drugs^28^. This process, called non-canonical MMR, is uncoupled from replication with the consequence that the DNA ends associated with the replication fork, which direct and activate classical MMR, are not automatically available. It was therefore proposed that non-canonical MMR may hijack nicks generated by BER^28^,^29^. Non-canonical MMR is associated with extensive gap formation^28^ and the prominent RPA foci observed here would therefore be compatible with non-canonical MMR. If the activating nicks for non-canonical MMR were intermediates generated by the BER pathway, we would also expect to see a dependency of UNG-1 for RPA filament generation and toxicity. However, no such dependency was observed. Hence, MMR does not hijack nicks generated through ordinary UNG-1-initiated BER. Although, we cannot exclude the possibility that MMR may hijack BER intermediates arising through processing by a cryptic, uncharacterized UDG activity^10^, the epistasis analyses suggested that the BER AP endonucleases EXO-3 and APN-1 function downstream of MutS. Non-redundant functions for APN-1 and EXO-3, as previously postulated^30^, were confirmed here, where only depletion of EXO-3 suppressed RPA-1 focus formation. These observations offer *in vivo* indications that EXO-3 provides nicked intermediates required for recruitment of EXO-1 and thereby activation of the MMR pathway. The *in vivo* substrates of EXO-3 and APN-1 remain unknown. As neither enzyme is known to incise undamaged DNA, it is likely to be that base damage is involved. As UNG-1-deficiency does not confer resistance, however, this base damage is likely not restricted to UNG-1 substrates. Approximately 77% of APN-1-depleted embryos still displayed prominent RPA-1-positive foci, indicating that the recently described nucleotide incision repair activity of APN-1 (ref. 31) contributes little to this function. RPA-1 focus formation in response to 5-FU depended on MutL, which distinguishes the pathway described here from the alternative mode for repair of oxidative DNA damage in which recruitment of low-fidelity DNA polymerase *η* depended on MutSα but not on MutLα (ref. 32). Consistently, EXO-1 was the main exonuclease required for RPA-1 focus formation, although the obliteration of RPA-1 focus formation in *exo-1* mutants upon APN-1 depletion suggested that the APN-1 3′–5′ exonuclease activity might contribute to DNA resection^31^. RPA-1 focus formation is used as an early marker of DDR activation. We were therefore interested to note that although RPA-1 foci formed in response to 5-FU in APN-1-depleted cells, they failed to mount a DNA-damage checkpoint response as measured by induction of CHK-1 phosphorylation. Hence, APN-1 functions downstream of RPA-1 filament formation to allow recruitment of the downstream factors required for checkpoint activation. Further experiments are required to pinpoint which activity of the multifaceted APN-1 enzyme is required for checkpoint activation, but the observation that some APN-1-depleted cells had extended RPA-1-positive tracts could point to a possible function of APN-1 in limiting MMR-mediated resection.

The observed APN-1-dependent CHK-1 phosphorylation further argues that autophagy was primarily induced as a consequence of DNA damage checkpoint activation resulting from processing the 5-FU-induced DNA damage via the pathway described. As autophagy is a cytoplasmic degradation pathway, it is not obvious how it is activated by DNA damage, but two observations allow us to present a model for autophagy activation in response to 5-FU. First, the appearance of VPS-34 foci in the nucleolus suggested that cargo in this organelle might be collected and targeted for degradation in 5-FU-treated cells. The degradation of RPS3 by autophagy in human cells supported this interpretation. Second, we showed that 5-FU primarily leads to global chromatin decompaction rather than DNA strand breaks. Our data therefore allow us to speculate that a relaxed chromatin landscape induced by MSH-6-initiated processing of 5-FU-induced DNA damage interferes with rDNA organization in the nucleoli leading to autophagy induction. Consistent with this model, the specific degradation of RPS3 by autophagy in human cells indicated that autophagy contributes to remove faulty or excess nucleolar proteins.

In summary, the data presented here allow us to propose a model for crosstalk between the BER and MMR pathways in eliciting DDR activation in response to 5-FU where the BER enzyme EXO-3 generates a nick required for MMR activation. Our data suggest that MSH-6-dependent processing of 5-FU-induced DNA damage either within or in the vicinity of the nucleoli is an event leading to toxicity and induction of autophagy. Together, our data strongly suggest that the role of autophagy in mediating DNA damage-induced cell death is more significant than previously anticipated.

## Methods

### *C. elegans* strains and culture conditions

*C. elegans* strains N2 Bristol, *msh-2(ok2410) I*, *mlh-1(ok1917)III* and *msh-6(pk2504) I* were obtained from *Caenorhabditis* Genetics Center. The *ung*-1(qa7600) III strain was generated previously^11^. The *exo-3 (tm4374)* and *exo-1 (tm1842)* were obtained from Shohei Mitani (Tokyo Women’s Medical University School of Medicine, Japan). All mutants are expected to be loss-of-function mutants, with the possible exception of the *mlh-1(ok1917)* mutant, which, if producing a stable protein, would give rise to a truncated protein of 521 aa (Supplementary Fig. S1). The *exo.3;msh-6* and *exo-3:mlh-1* double mutants were generated for this study. Reporter strains has the following genotypes: *buEx070[plgg-1::GFP::LGG-1+rol-6(su1006)]* and *bec-1(ok691)*; *swEx520[pbec-1::BEC-1::GFP+rol-6(su1006)]* and *Ex[atf-2::gfp+unc-119(+)]*; *unc-119(e2497)*, *him-5(e1490)V*. *adIs2122[lgg-1::GFP+rol-6(su1006)]* and *atm-1(gk186)*; *adIs2122[lgg-1::GFP+rol-6(su1006)]*, which were kind gifts from Natascia Ventura (Leibniz Research Institute for Environmental Medicine, Düsseldorf, Germany). The *atl-1(tm853) IV/nT1[unc-?(n754) let-? qIs50] (IV;V)* was a gift from Simon Boulton (Cancer Research UK, London Research Institutes, South Mimms, UK). An *Ex[MSH-2::GFP+unc-119(+)]; unc-119(e2497* was generated for this study. Briefly, the *msh-2* coding region was amplified using the reverse primer 5′-TTTCCCGGGacaaggctgagaatggcttg-3′ and forward primer 5′-caagccattctcagccttgtAAACCCGGG-3′, and cloned into the pPD95.77 vector into the *Sma*I and *Xba*I sites. The construct was cobombarded (with a plasmid containing *unc-119(+)*) into *unc-119(−)* mutant animals. The non-Unc progenies were selected and screened for GFP expression. Worms were cultured and maintained at 20 °C using standard procedures, using OP50 as the food source. For experiments using the *atl-1(tm853) IV/nT1[unc-?(n754) let-? qIs50] (IV;V)* strain, *unc* and non*-unc* young adults were exposed to 5-FU, embryos fixed and stained with anti-VPS-34 antibodies. Images of non*-unc* GFP-negative homozygous *atl-1(tm853)* mutants are presented. For RNAi experiments, worms were maintained for three generations on Nematode Growth Medium (NGM) plates containing 2 mM IPTG (isopropyl β-D-1-thiogalactopyranoside) seeded with *Escherichia coli* HT115(DE3) expressing RNAi constructs in the *pL4440* feeding vector. The culture condition was 20 °C.

### Chemicals and antibodies

5-FU, 5-fluoro-2′-deoxyuridine, methylmethanesulphonate, Mx and 3-MA were from Sigma (Oslo, Norway). The following commercially available antibodies were used: LC3, Ulk1, poly (ADP-ribose) polymerase, Caspase-3 and MSH-6 (Cell Signaling Technology); RPS3 and actin (Abcam); β-actin (Sigma-Aldrich); MSH-2, P-Histone 3(pSer10), P-CHK-1 (pSer345) (Santa Cruz Inc.); anti-Cdk1 (pTyr15) (VWR); and GFP (Roche). Antibodies directed against the following *C. elegans* proteins were kinds gifts: VPS-34 from Fritz Muller (University of Fribourg); RPA-1 and RAD-51 from Anton Gartner (University of Dundee); and LMN-1 from Yosef Gruenbaum (The Hebrew University of Jerusalem, Israel). RPA-1 from Hyeon Sook Koo (Yonsei University, Seoul, Republic of Korea) and H1X.101 from Monika A Jedrusik (Max Planck Institute for Biophysical Chemistry, Goettingen, Germany). Secondary antibodies were Cy3-conjugated anti-rabbit (Sigma-Aldrich), Alexa 488-conjugated anti-rat and anti-mouse, Alexa Fluor 555-conjugated anti-rabbit and anti-rat (Invitrogen), enhanced chemiluminescence (ECL) anti-rabbit IgG horseradish peroxidase (HRP)-linked whole antibody (GE Healthcare) and anti-mouse IgG HRP-linked (Santa Cruz).

### *C. elegans* toxicity assays

Standard *C. elegans* toxicity assays were performed after feeding OP50 to N2 or mutant strains on *E. coli* or after feeding on *E. coli* HT115(DE3) expressing RNAi. Three stage-4 larvae (L4) were transferred to seeded NGM plates containing 5-FU (0–4 μM) and 2 mM IPTG, and allowed to lay eggs for 28 h. The number of embryos laid was scored after the removal of the hermaphrodite. Survival was scored as the fraction (%) of offspring that developed into adults after 72 h and presented as the mean±s.d. from four independent replicates with at least 150 animals per data point.

### *C. elegans* autophagy

A transgenic reporter strain expressing functional LGG-1 in fusion with GFP (GFP::LGG-1) was used to monitor autophagy induction. Young adults grown on RNAi-expressing food were exposed to 100 μM 5-FU along with RNAi food for 24 h. Worms were anaesthetized with 10 mM levamisol (Sigma) and mounted on 4% agarose pads. Induction of autophagy was monitored under a Zeiss LSM-510 META Confocal microscope with × 63 Plan-Apochromat 1.4 numerical aperture (NA) objective. The number of worms having GFP-positive foci in embryos was scored in 10–60 worms per condition in four independent experiments. To monitor the induction of autophagy in seam cells, L1- to L2-stage larvae were exposed to 100 μM 5-FU for 24 h before scoring the average number of GFP-positive puncta in the seam cells at the L3 stage under a Zeiss Axiovert 200M inverted microscope with × 100 Plan-Apochromat 1.45 NA objective and standard epifluorescence filters. At least four to eight seam cells were observed in each worm in three independent experiments. Expression of the ATF-2 transcription factor in fusion with GFP (ATF-2::GFP) was measured using a transgenic reporter strain in 5-FU-treated hermaphrodites fed control, *msh-2, msh-6*, or *exo-3* RNAi. ATF-2::GFP expression was scored in at least 150 animals per condition.

### *C. elegans* immunofluorescence

For immunohistochemistry, embryos or dissected germlines were collected 24 h after treating synchronized L4 hermaphrodites with 125 Gy ionizing radiation or 100 μM 5-FU.

Embryos were placed in 1 × PBS buffer on polylysine-coated slides (Thermo Scientific), covered with coverslips and frozen on dry ice for 20 min. The embryos were fixed in 1:1 acetone:methanol for 10 min at −20 °C, washed in PBS-T (1 × PBS, 0.1% Tween-20) for 5 min, followed by 30 min incubation with image-IT FX signal enhancer (Invitrogen) and 30 min blocking in PBS-TB (1 × PBS, 0.1% Tween-20, 0.5% BSA). The slides were incubated with primary antibody overnight at 4 °C, washed three times 10 min in PBS-T, followed by incubation with the secondary antibody at room temperature for 2 h. Finally, the embryos were washed three times for 10 min in PBS-T and mounted with 7 μl mounting solution containing VECTASHIELD (Vector Lab) and 0.5 μg ml^−1^ DAPI (Sigma). Germlines were dissected on polylysine-coated slides in egg buffer (25 mM HEPES, pH 7.4, 0.118 M NaCl, 48 mM KCl, 2 mM CaCl_2_, 2 mM Mg Cl_2_) supplemented with 0.1% Tween-20 and 0.2 mM levamisol. Germlines were fixed in 4% formaldehyde for 5 min at room temperature and freeze-cracked in liquid nitrogen before further processing, as described above, for embryos. Primary antibodies were used at the following dilutions: RAD-51 (1/200), RPA-1 (1/200), Histone 3 (pSer10) (1/400) and Cdk1 (pTyr15) (1/100). The following secondary antibodies were used for detection: Cy3-conjugated anti-rabbit at 1/10,000 and 1/1,000 for the detection of RAD-51 and Cdk1, respectively, and Alexa 488-conjugated anti-rat at 1/1,000. Primary antibodies were used at the following dilutions; VPS-34 (1:200), RAD-51 (1:100), LMN-1 (1:400) and pCHK1 (1/50). Anti RPA-1 antibodies were used at (1:200) or (1/1,000) (from Drs Gartner and Koo, respectively). The following secondary antibodies were used for detection: Alexa Fluor 555-conjugated anti-rabbit at 1/1,500 for detection of VPS-34, Rad-51, LMN-1 and pCHK1. Alexa Fluor 555 anti-rat at 1/1,500 to detect RPA-1 (Gartner), and Alexa Fluor 488-conjugated anti-mouse at 1/1,000 to detect RPA-1 (Koo). The slides were imaged under a Zeiss LSM-510 META^MK14^ Confocal microscope with × 63 Plan-Apochromat 1.4 NA objective.

### *C. elegans* nucleolar stress

Induction of nucleolar stress was monitored as previously described^33^. Briefly, embryos were dissected out of 5-FU-treated hermaphrodites and observed continuously from the three-cell through the four-cell stage under differential interference contrast in a Zeiss Axiovert 200M inverted microscope with × 100 Plan-Apochromat 1.45 NA objective. A fraction of four-cell stage embryos with visible nucleoli was scored in three independent experiments comprising a minimum of eight embryos per experiment.

### *C. elegans* chromatin decompaction

To examine the chromatin decompaction state after 5-FU treatment, dissected embryos were immunostained with α-LMN-1 as described above to visualize the nuclear membrane. The maximum nuclear diameter was measured in 4-cell embryos in 15–32 embryos per condition after identification of the appropriate focal plane from a *z*-stack under a Zeiss LSM-510 META^MK14^ Confocal microscope with × 63 Plan-Apochromat 1.4 NA objective.

### Human cells and transfection

Human osteosarcoma U2OS cells were grown under 5% CO_2_ in DMEM media supplemented with 10% FCS, 100 U ml^−1^ penicillin and 100 μg ml^−1^ streptomycin (all from Gibco). Cells were transfected with siRNA oligos in the absence of antibiotics and serum 1 day after seeding into six-well plates (1.5 × 10^5^ cells per well), using 1 ml transfection solution containing 0.64 μl Lipofectamine RNAiMAX (Invitrogen) and a final concentration of 10 nM siRNA according to the manufacturer’s instructions. After 5 h, the cells received complete growth medium and were cultured for 24 h before addition of the drugs indicated in the figure legends. The siRNA oligos had the following sequences (sense strand): Ulk1-a, 5′-CCACGCAGGUGCAGAACUA-3′ (Dharmacon); Ulk1-b, 5′-UCACUGACCUGCUCCUUAA-3′ (Dharmacon); MSH-2, 5′-CGUCGAUUCCCAGAUCUUA-3′ (GE Healthcare Ambion). Control cells were transfected with a non-targeting control duplex (Dharmacon).

### Human cell immunofluorescence

U2OS cells were seeded in Lab-Tek II Chambered Coverglass (Nunc) 1 day before the experiments were performed and treated with 5-FU (10 μM) or Mx (1 mM) for the indicated times. The cells were then fixed in 100% methanol for 10 min and blocked in 5% FCS before incubation with anti-LC3 antibody (Clone 5F10, Nanotools), followed by incubation with Alexa488-labelled donkey anti-mouse antibody (Molecular Probes). The nuclei were stained with DAPI (1 μg ml^−1^) in PBS. Pictures were taken using a Cell Observer microscope (Zeiss) equipped with a × 40 objective. The number of LC3 spots per cell was quantified from 200 to 1,600 cells per condition in each experiment. Four independent experiments were performed and the mean number of LC3 spots per cell was normalized to that of untreated control cells.

### Quantitative real-time reverse transcriptase–PCR

Transcriptional activation of *C. elegans ced-13* and *egl-1* was measured in synchronized L4 hermaphrodites treated with 100 μM 5-FU for 24 h. For total RNA extraction, worms were disrupted in TRIZOL with 0.7 mm zirconia/silica beads (Biospec Products) using a Mini-Beadbeater 8 (Biospec Products) at maximum speed for 30 s. Complementary DNA synthesis was performed using iScript cDNA synthesis kit from Bio-Rad, according to the manufacturer’s instructions. Quantitative PCR was performed with SYBR Green supermix (Bio-Rad) starting at 95 °C for 30 s, followed by 50 cycles at 95 °C for 30 s, 55 °C for 30 s and 72 °C for 30 s. Transcript levels were normalized to an internal tubulin (*tbg-1*) control. Primers with the following sequences were used: for *egl-1* (5′-CCTCAACCTCTTCGGATCTT-3′) and (5′-TGCTGATCTCAGAGTCATCAA-3′); for *ced-13* (5′-GCTCCCTGTTTATCACTTCTC-3′) and (5′-CTGGCATACGTCTTGAATCC-3′); and for *tbg-1* (5′-AAGATCTATTGTTCTACCAGGC-3′) and (5′-CTTGAACTTCTTGTCCTTGAC-3′). RNA isolated 24 h after irradiation with ionizing radiation (125 Gy) was included as a positive control. As a control of RNAi efficiency, mRNA expression levels were measured in embryos collected after three generations of feeding N2 animals on *E. coli* expressing RNAi, targeting the indicated genes. The primers were as follows (sense strand): *msh-6* forward: 5′-GATTTGGGAAGTGCTTCGTC-3′, reverse: 5′-TGCAGTCGTTGTGTCAATCA-3′; *msh-2* forward: 5′-GAGTGGAGGAAAAGACGAAG-3′, reverse: 5′-CATTTGTTGAGAATTGTCGGTTG-3′; *mlh-1* forward: 5′-GAGGAGAAGCTCTTGCATCG-3′, reverse: 5′-GCGGTCATTTTTCCGTCTAA-3′; *apn-1* forward: 5′-ACCGGCTATCAGGAAATTGA-3′, reverse: 5′-CCGACTCTTCCTCTTCTTTCA-3′; *exo-3* forward: 5′-GGAGGAGACGTTTAAGAACTAC-3′, reverse: 5′-GCTCCGATGAAGGTTCACA-3′; *bec-1* forward: 5′-GCGAAACAGTTATCACAGAAGC-3′, reverse: 5′-GAGCGTCAGAGCAATCATTAC-3′; and *atg-7* forward: 5′-ATGGCCACGTTTGTTCCC-3′, reverse: 5′-CTTCGGTTTGATGAAGCGAT-3′. For isolation of total RNA from human cells, the RNeasy Plus Mini Kit (QIAGEN) was used according to the manufacturer’s instructions. Total RNA (0.8 μg) was used for cDNA synthesis using the iScript cDNA Synthesis Kit (Bio-Rad). The real-time PCR analysis was run on a CFX96 Real-time PCR Detection System (Bio-Rad) using SsoFast EvaGreen Supermix (Bio-Rad) and prevalidated Quanti-Tect Primer Assays (QIAGEN). The cycling conditions were 95 °C for 1 min, followed by 40 cycles of 95 °C for 5 s, 55 °C for 5 s and 72 °C for 5 s. Relative quantities of Exo-1 or RPS3 transcripts were determined using the regression analysis method provided in the CFX Manager Software (Bio-Rad). Transcript quantities were normalized to the relative quantity of the house-keeping genes TBP (TATA-box binding protein) and SDHA (succinate dehydrogenase complex, subunit A, flavoprotein) for each condition. The following prevalidated Quanti-Tect Primer Assays were used: Hs_RPS3_2_SG, Hs_EXO1_1_SG, Hs_TBP_1_SG and Hs_SDHA_2_SG.

### Western blot analyses

Synchronized young GFP::LGG-1-expressing adults were cultured for 24 h on 10 cm OP50-seeded plates with no treatment or in the presence of 100 μM 5-FU. 3-MA (10 mM) was added after 20 h to inhibit autophagy. Embryos were collected by bleaching, washed and resuspended in worm lysis buffer (40 mM Tris-HCl, pH 7.4, 150 mM NaCl, 0.05%, NP-40, 2 mM EDTA, 1 tablet protease inhibitor from Roche) from N2, *msh-6(pk2504)*, *msh-2(ok2410)* and *MSH-2::GFP*. After addition of 1.0 mm zirconia beads (BioSpec product), the embryos were disrupted in a Mini-Beadbeater 8 (BioSpec Products) as described previously^33^. Protein concentrations were determined using the Bradford Reagent (Bio-Rad) and the extracts were separated on criterion 12.5 and 7.5% precast gels (Bio-Rad), followed by electroblotting to Immobilon-P membranes (Millipore). The blots were probed with primary antibodies (GFP (1:1,000), actin (1:1000) and MSH-6 (1:1,200)) at 4 °C overnight, washed, detected with anti-mouse IgG HRP-linked (Amersham) and anti-rabbit Ig-G HRP (Santa Cruz), and visualized using Supersignal west pico and west femto (Thermo Scientific).

For detection of histone 1 × expression, protein extracts were prepared by boiling embryos in nematode solubilizing buffer^34^ (0.3% ethanolamine, 2 mM EDTA, 1 mM phenylmethylsulphonyl fluoride, 5 mM dithiothreitol and 1 × protease inhibitor) for 25–50 s and immediately added SDS buffer along with reducing agent from the Nu-Page system (Invitrogen). The crude extract was separated in 12% Novex bis tris gel (Invitrogen). The blots were probed with anti H1X.101 (1/1,000)^35^ and actin (1/1,000) antibodies, detected with ECL anti-rabbit Ig-G HRP (GE Healthcare) antibody at 1/25,000 dilution and visualized using Supersignal west pico and west femto (Thermo Scientific).

For preparation of human whole-cell extracts, U2OS cells were washed in cold PBS, lysed in RIPA buffer supplemented with a mixture of protease inhibitors (Roche) and scraped. After a brief sonication, the lysates were cleared and the protein concentrations determined by the BCA Assay (Pierce). Fifteen micrograms of protein per sample was loaded and resolved on 4–20% gradient gels (Bio-Rad) followed by electroblotting to Immobilon-P membranes (Millipore). The blots were probed with specific antibodies, which were detected using standard ECL reagents. The band signal intensities were quantified by the Quantity One software (Bio-Rad) and normalized to those of actin. Full blots can be found in Supplementary Fig. S8.

### Statistical analysis

Mean values±s.e.m. were calculated for each condition. The statistical significance of the differences was determined by paired Student’s *t*-test. *P<0.05; **P<0.01; ***P<0.001 were considered to be statistically significant.

## Additional information

**How to cite this article:** SenGupta, T. *et al.* Base excision repair AP endonucleases and mismatch repair act together to induce checkpoint-mediated autophagy. *Nat. Commun.* 4:2674 doi: 10.1038/ncomms3674 (2013).

## Supplementary Material

Supplementary Information Supplementary Figures S1-S8 and Supplementary Tables S1-S4
