# Publisher Correction: Base excision repair AP endonucleases and mismatch repair act together to induce checkpoint-mediated autophagy

**DOI:** 10.1038/ncomms16206

**Published:** 2018-03-29

**Authors:** Tanima SenGupta, Maria Lyngaas Torgersen, Henok Kassahun, Tibor Vellai, Anne Simonsen, Hilde Nilsen

Nature Communications 4: Article number: 2674; DOI: 10.1038/ncomms3674 (2013); Published online 10 24 2013; Updated 03 30 2018

The original HTML version of this Article had an incorrect article number of 2578; it should have been 2674. This has now been corrected in the HTML; the PDF version of the Article was correct from the time of publication.

