# Supplementary material for: Base excision repair AP endonucleases and mismatch repair act together to induce checkpoint-mediated autophagy
Source: Nat Commun. 2013 Oct 24;4:2674. doi: 10.1038/ncomms3674 (PMC3826653; doi:10.1038/ncomms3674)
Supplement: Supplementary Information — Supplementary Figures S1-S8 and Supplementary Tables S1-S4 [file ncomms3674-s1.pdf]

Base Excision Repair AP-endonucleases and Mismatch Repair act together to Induce Checkpoint Mediated Autophagy

Tanima SenGupta[1], Maria Lyngaas Torgersen[2], Henok Kassahun[1], Tibor Vellai[3], Anne Simonsen[2] and Hilde Nilsen[1]*

SUPPLEMENTARY INFORMATION LISTING

- **Supplementary Figure S1** Mutants
- **Supplementary Figure S2** BER and MMR mediated toxicity of 5-FU
- **Supplementary Figure S3** DNA damage checkpoint activation in the *C. elegans* germline and embryos in response to 5-FU.
- **Supplementary Figure S4** Global chromatin decondensation in 5-FU treated embryos.
- **Supplementary Figure S5** 5-FU induced autophagy in *C. elegans* embryos.
- **Supplementary Figure S6** RPS3 is degraded by autophagy in 5-FU treated U2OS cells.
- **Supplementary Figure S7** Depletion of DNA repair pathways other then BER and MMR does not lead to 5-FU resistance
- **Supplementary Figure S8** Original western blots
- **Supplementary Table S1** *5-FU induced RPA-1 foci in embryos*
- **Supplementary Table S2** *5-FU induced RPA-1 foci in embryos in mutants*
- **Supplementary Table S3** *Fraction of embryos with RAD-51 foci*
- **Supplementary Table S4** *5-FU induced autophagy in embryos*

Supplementary Fig S1

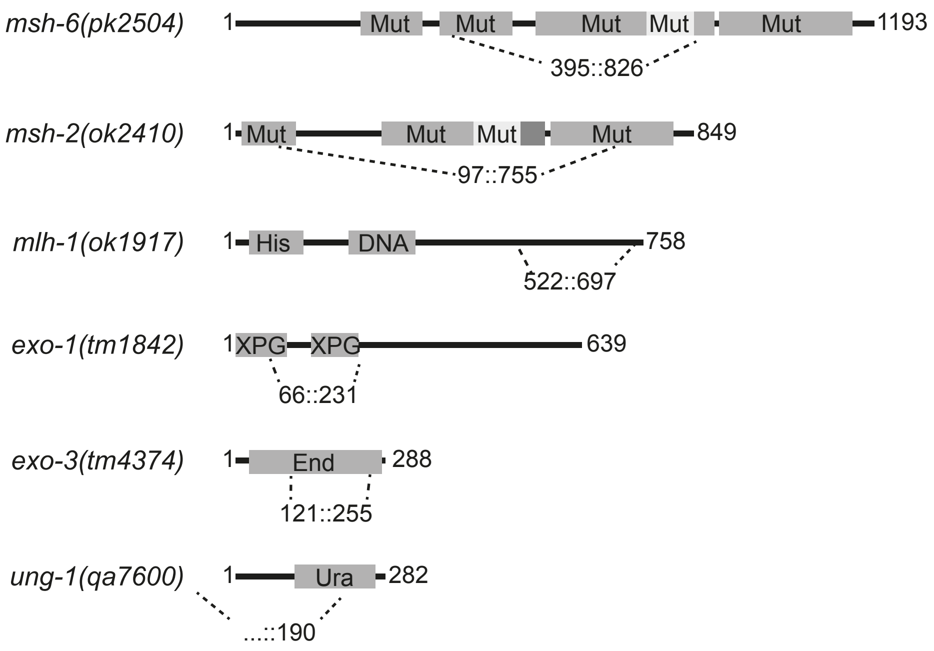

Supplementary Figure S1 Mutants. Cartoons showing the architecture of the indicated DNA repair proteins with conserved pfam domains important for DNA binding or activity as given in Wormbase (www.wormbase.org). The deletions expected from the mutant alleles used in this study are indicated with amino acid numbers. All deletion mutations are expected to be loss-of-function alleles, with the possible exception of the *mlh-1(ok1917)* allele that might lead to the production of a truncated protein.

Supplementary Fig S2

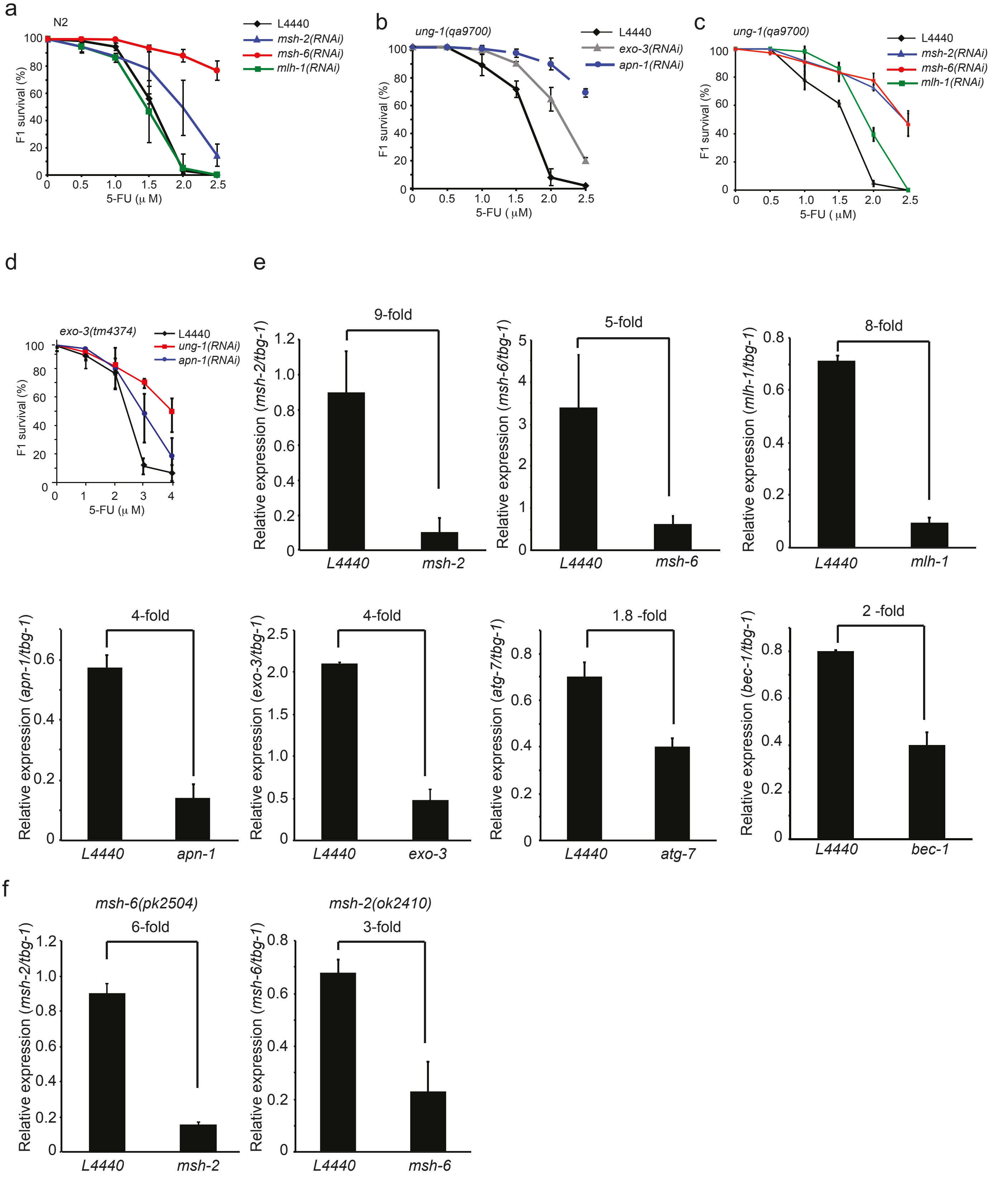

Supplementary Figure S2 BER and MMR mediated toxicity of 5-FU. (**a**) F1 survival following depletion of MSH-6 (red circles), and MSH-2 (blue triangles) and MLH-1 (green squares) in N2. (**b**) F1 survival following depletion of the AP-endonucleases EXO-3 (grey triangles) and APN-1 (blue circles) in the ung-1(qa7600) mutant. (**c**) F1 survival following the depletion of MSH-6 (red circles), and MSH-2 (blue triangles) and MLH-1 (green squares) in *ung-1(qa7600)*. (**d**) F1 survival after depletion on APN-1 (blue circles) and UNG-1 (red squares) in *exo-3(tm4374)* mutant animals. (**a-d**) The survival curves show the mean ± s.d. for each data point from three independent experiments. (**e**) qRT-PCR determination of mRNA expression levels of the indicated genes after RNAi depletion in N2. (**f**) qRT-PCR determination of mRNA expression levels after RNAi depletion in the mutants indicated. (**e**, **f**) Bar graphs present mean ± s.d. from three independent experiments.

Supplementary Fig S3

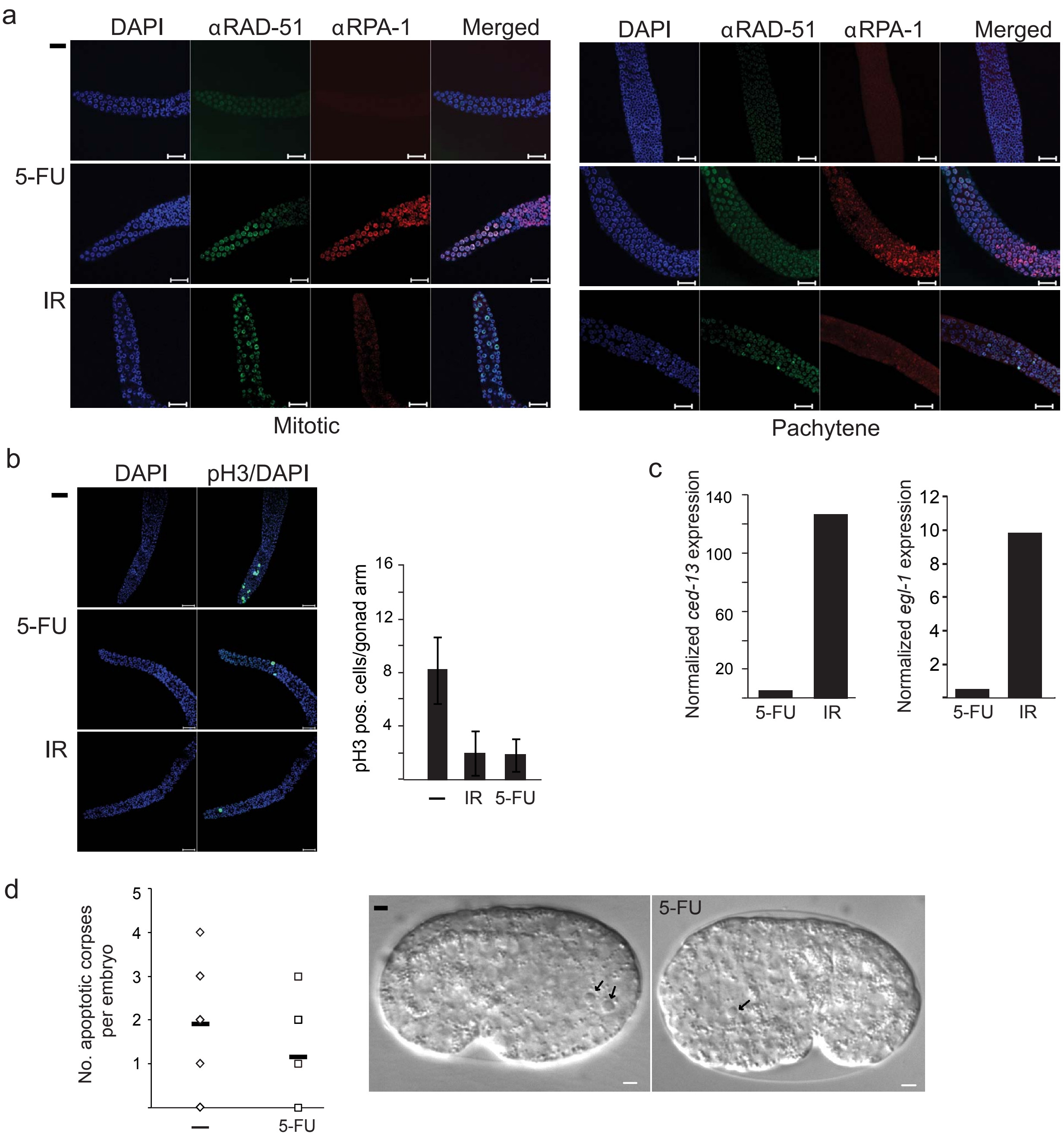

Supplementary Figure S3 DNA damage checkpoint activation in the *C. elegans* germline and embryos in response to 5-FU. (**a**) All cytological markers for the activation of DNA damage response signaling (DDR) were similar in germlines treated with 5-FU and ionizing radiation (IR). Immunostaining showing the accumulation of anti-RPA-1 and anti-RAD-51 positive foci in the mitotic (left panel) and pachytene (right panel) regions of the germline, following 5-FU treatment. Immunostaining in untreated germlines (upper panel) and after ionizing radiation (125 Gy, IR; lower panel) are shown as negative and positive controls, respectively. DNA was stained with 4',6-diamidino-2-phenylindole (DAPI) (20 μm scale bars). (**b**) Activation of cell-cycle arrest in response to 5-FU and IR illustrated by staining with antibodies recognizing phosphorylated H3 (pH3) in the germline mitotic region (10 μm scale bars). The number of pH3-positive nuclei per gonad arm was counted in 10 animals per treatment group in three independent experiments and is given as an average ± s.d. (**c**) Transcriptional induction of the CEP-1 responsive genes *ced-13* and *egl-1* in 5-FU treated and irradiated worms. mRNA levels were measured by qRT-PCR in triplicate and normalized to an internal tubulin (*tbg-1*) control. (**d**) The number of apoptotic corpses in embryos induced by 5-FU was scored in a minimum of 10 embryos under DIC microscopy (scale bar = 5 μm). The average number of corpses is indicated by the black horizontal bar.

Supplementary Fig S4

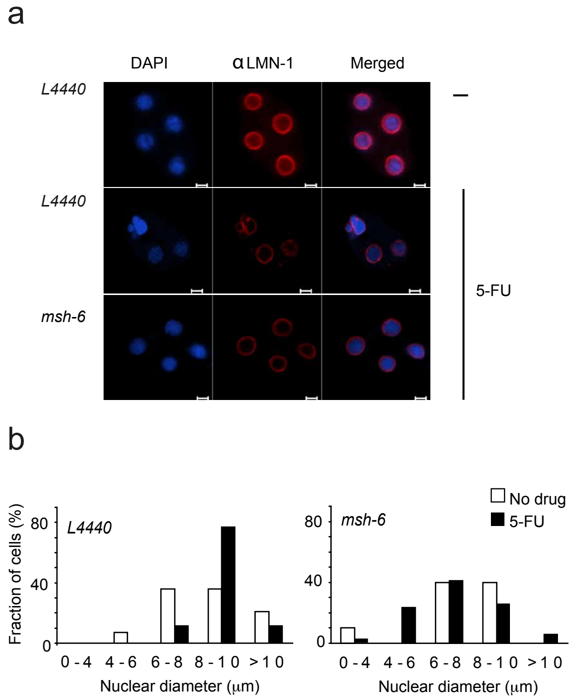

Supplementary Figure S4 Global chromatin decompaction in 5-FU treated embryos. (**a**) Visualization of the nuclear envelope by confocal microscopy after immunostaining with anti-LMN-1 antibodies in wild-type embryos grown on bacteria expressing empty vector control (L4440) or *msh-6* RNAi. DNA was counterstained with DAPI (scale bars in IF images = 5 μm). (**b**) The maximum nuclear diameter of a minimum of 15 nuclei were identified from z-stacks and measured using the LSM-software. The size distribution was plotted as the fraction (%) of nuclei of the indicated size intervals in the absence (white bars) or presence (black bars) of 5-FU. The maximum nuclear diameter increased from 8.2 μm to 9.0 μm (Student t-test, $p<0.05$) by 5-FU treatment. The maximum nuclear diameter was unaffected by 5-FU in MSH-6 depleted embryos (maximum nuclear diameter of 7.6 and 7.3 μm in untreated and treated embryos, respectively (Student´s t-test $p = 0.3$).

Supplementary Fig S5

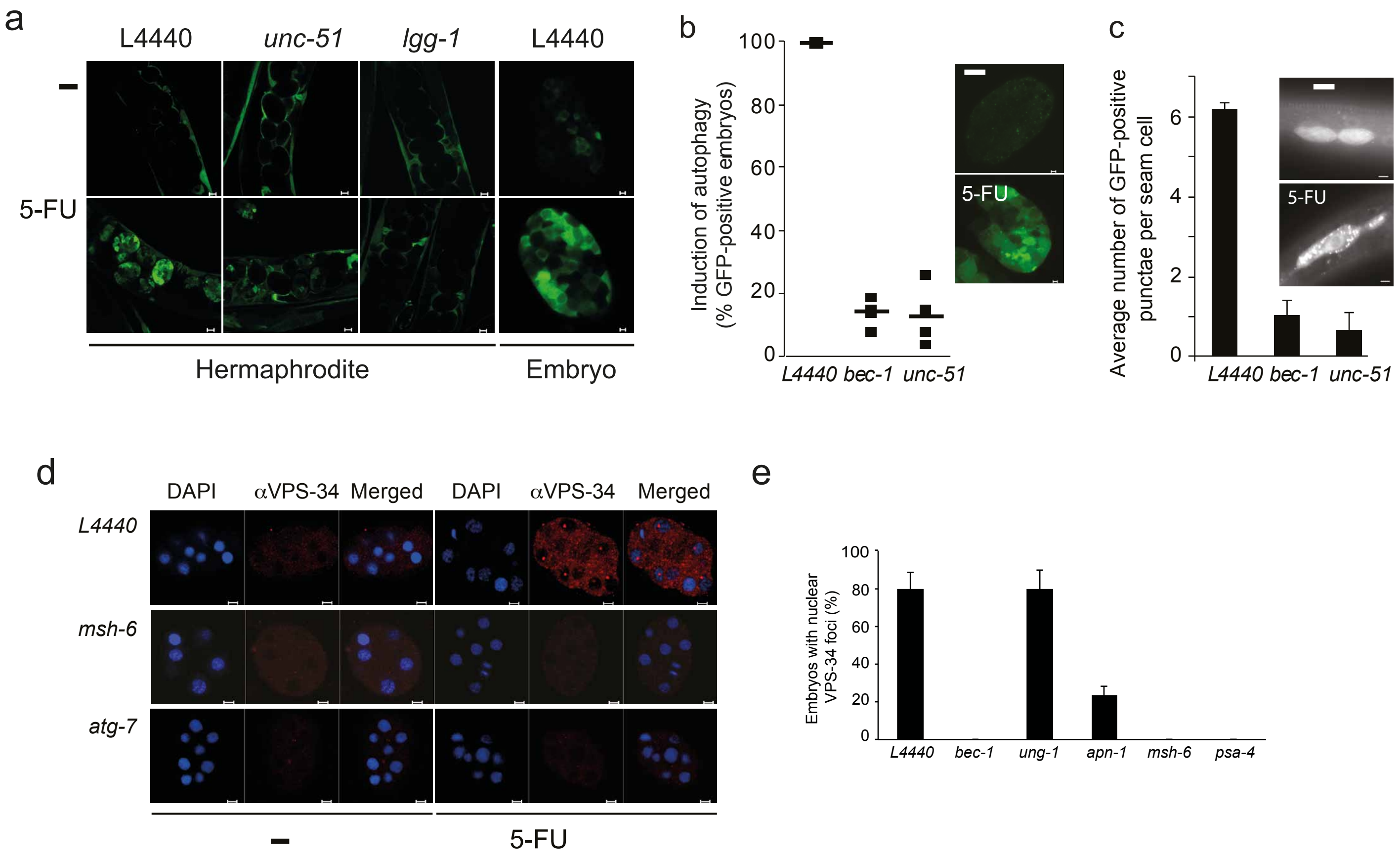

Supplementary Figure S5 5-FU induced autophagy in *C. elegans* embryos. (**a**) 5-FU-induced autophagy monitored in the BEC-1::GFP translational reporter strain grown for three generations on bacteria expressing empty vector control (L4440) or RNAi targeting the indicated autophagy genes (scale bar = 10 μm). Embryos show increased GFP expression levels and distinct GFP-positive puncta (scale bar = 2 μm).
(**b**) Confocal images showing excessive GFP expression and GFP positive puncta in dissected embryos (2 μm scale bar) in response to 5-FU in an GFP::LGG-1 translational fusion reporter strain fed control or RNAi targeting the indicated core autophagy genes. The fraction (as % of the untreated control) of hermaphrodites having embryos with excessive GFP expression following 5-FU treatment was quantified in each experiment (filled square) and the mean value indicated (line) are presented.
(**c**) Induction of autophagy in between four to eight L3 stage seam cells per animal was scored and presented as the average number of GFP-positive puncta per seam cell (5 μm scale bar). (**b**, **c**) 10 to 40 animals were scored for each condition in four independent experiments. (**d**) Confocal images showing induction of autophagy by immunostaining with anti-VPS-34 antibodies in embryos dissected out from N2 wild-type worms grown on bacteria expressing the indicated RNAi (scale bar = 5 μm). VPS-34 accumulation depended on ATG-7, confirming that the accumulation is part of the autophagic programme. (**e**) Quantification of the fraction (as % of total) of embryos with nucleolar VPS-34 foci. (**c**, **e**) Bar graphs present mean ± s.d. from three independent experiments.

Supplementary Fig S6

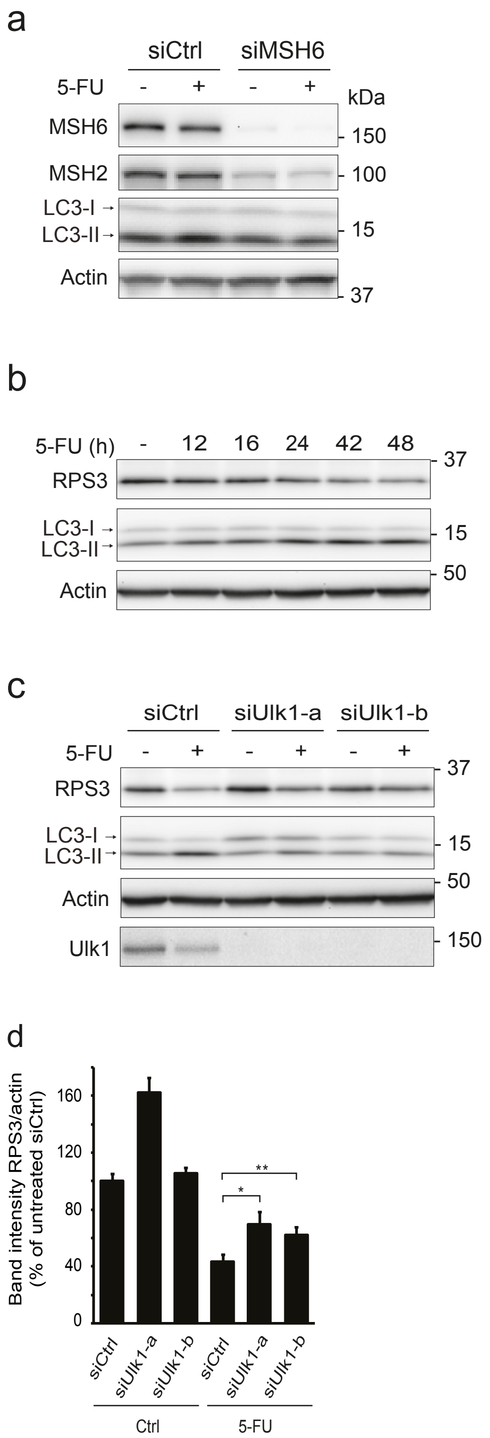

Supplementary Figure S6 RPS3 is degraded by autophagy in 5-FU treated U2OS cells. (**a**) Western blot showing effective knockdown of MSH6 using specific siRNA, accompanied by loss of MSH2 stability in U2OS cells. (**b**) Western blot showing progressive reduction in RPS3 level and increased LC3 II levels after 5-FU treatment. (**c**) The 5-FU induced reduction in RPS3 protein level is partially rescued upon knockdown of Ulk1. Western blot of lysates from control or 5-FU treated U2OS cell transfected with siRNA oligos against Ulk1 or non-targeting control. (**d**) Normalized RPS3 protein levels upon 5-FU-treatment of control- or siUlk1-transfected U2OS cells presented as the mean ± SEM, from at least 3 independent experiments (Student´s t-test * $p < 0.03$, ** $p < 0.001$).

Supplementary Fig S7

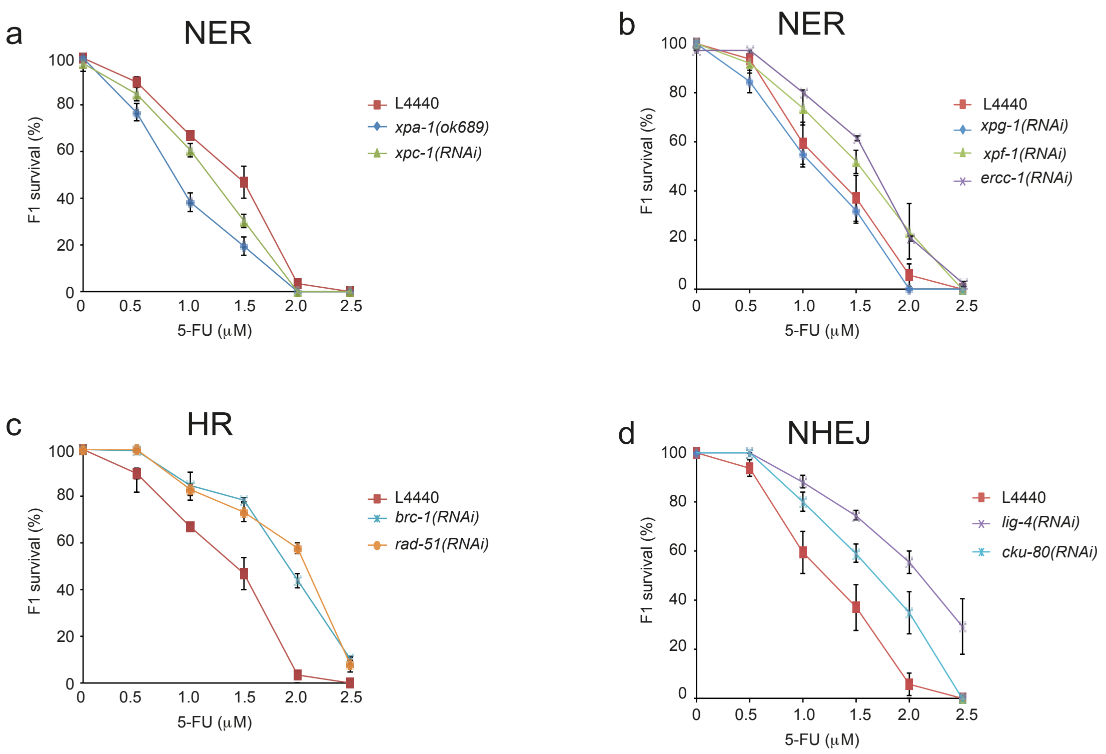

Supplementary Figure S7 Depletion of DNA repair pathways other than BER and MMR does not lead to 5-FU resistance. (**a**) F1 survival experiments in the Nucleotide Excision Repair (NER) mutant *xpa-1(ok698)*, or survival of N2 animals grown for three generations on food expressing control RNAi (L4440) or RNAi directed against the NER DNA damage recognition protein XPC-1. (**b**) F1 survival of N2 animals after depletion of the NER structure-specific endonucleases XPG-1, XPF-1, or ERCC-1. (**c**) F1 surivial of N2 animals after depletion of Homologous Recombination (HR) repair proteins BRC-1 or RAD-51. (**d**) F1 survival following depletion of Non-Homologous End-Joining (NHEJ) proteins LIG-4 or CKU-80. (**a-d**) The survival curves show the mean ± s.d. for each data point from three independent experiments.

Supplementary Fig S8

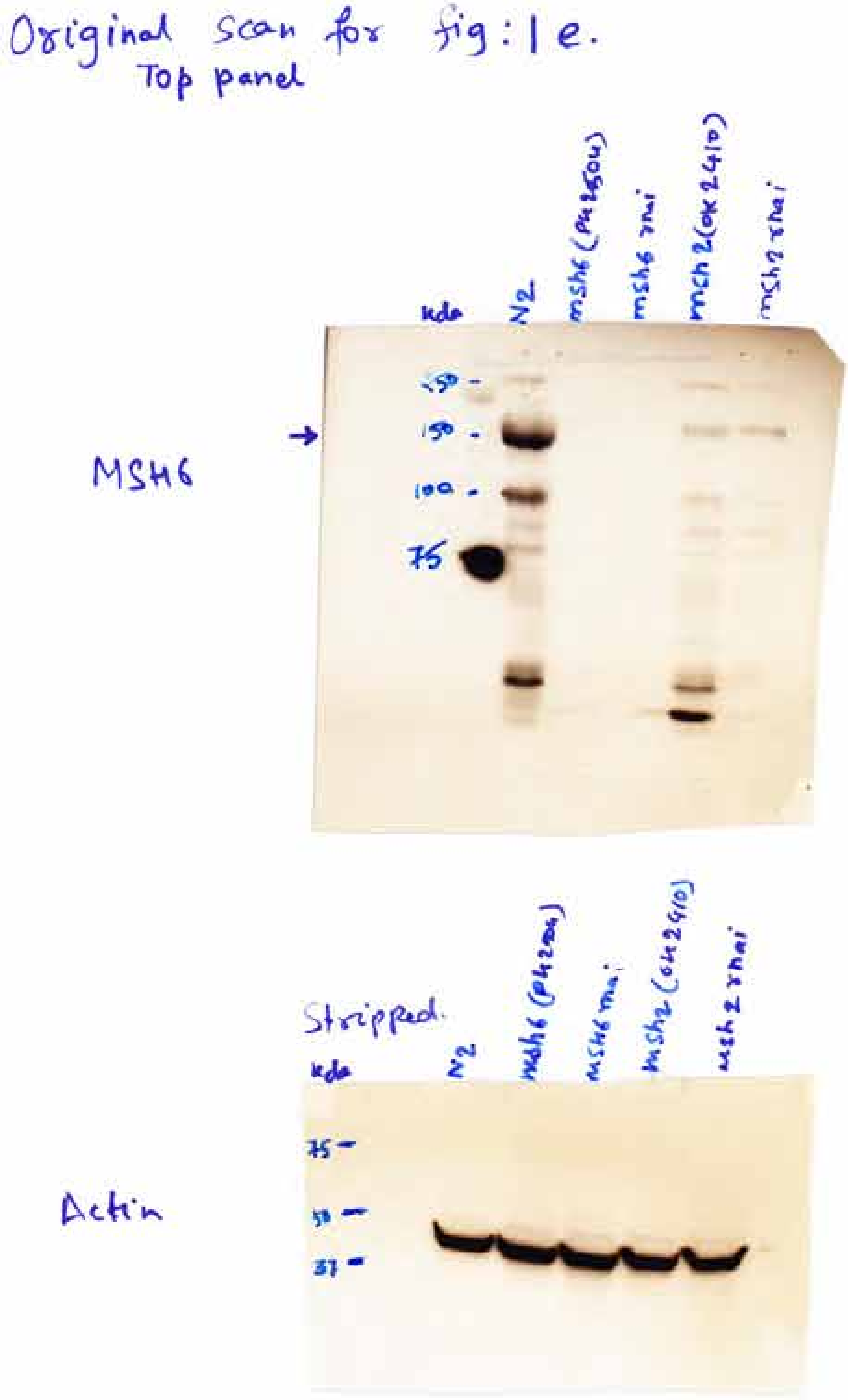

Original Scan for fig: 1 e
Lower panel

GFP antibody.

121kda is MSH-2::GFP band

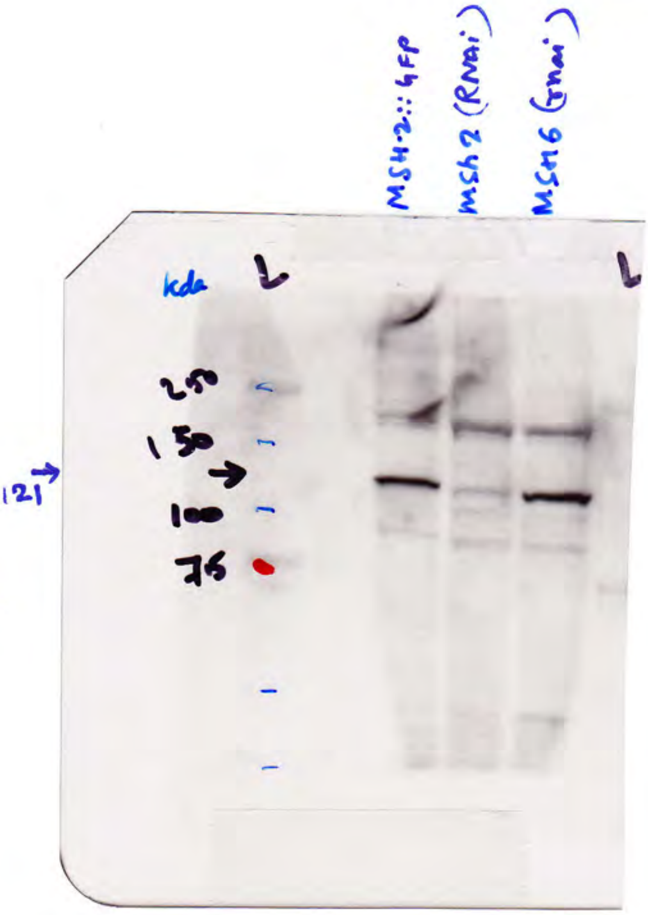

Stripped

Actin

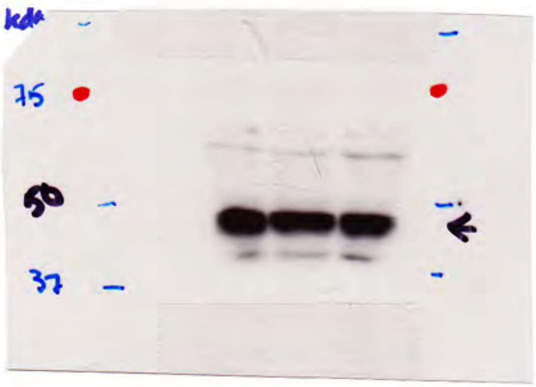

Original Scan for fig 2 f.

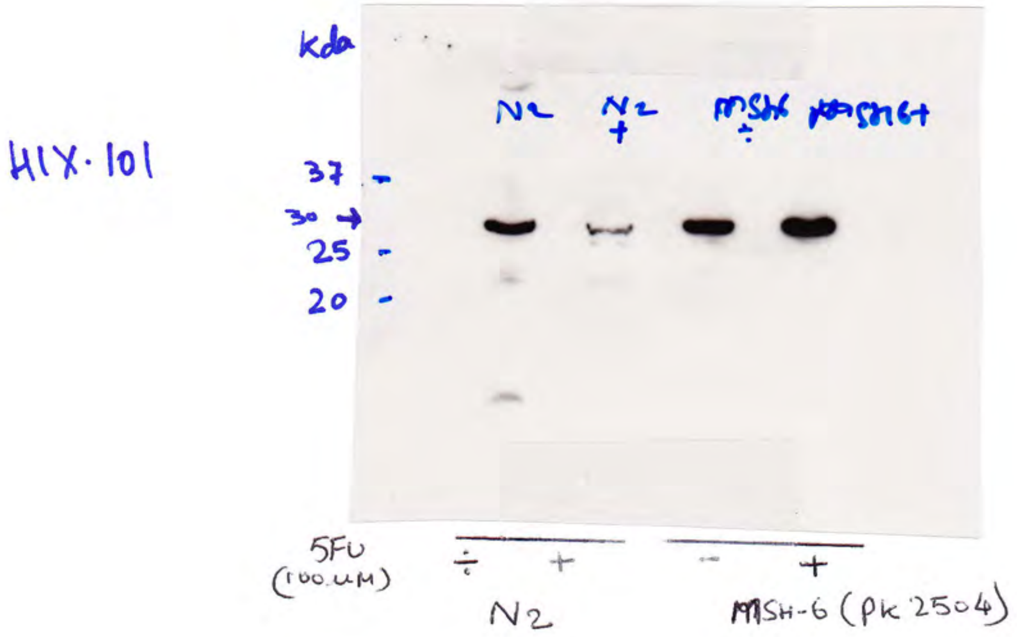

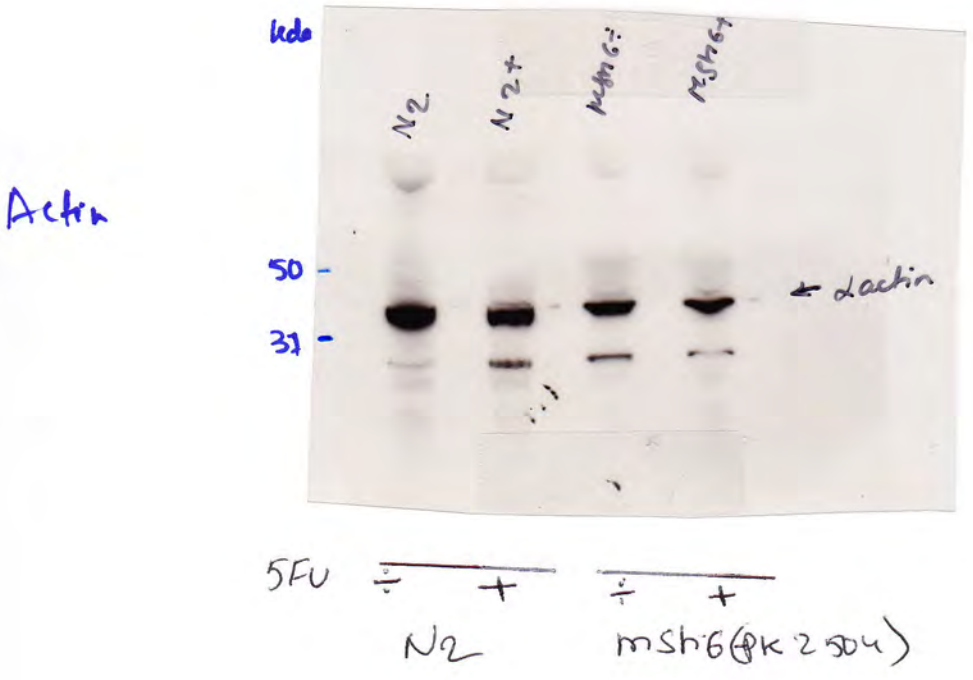

Original Scan for fig 3c

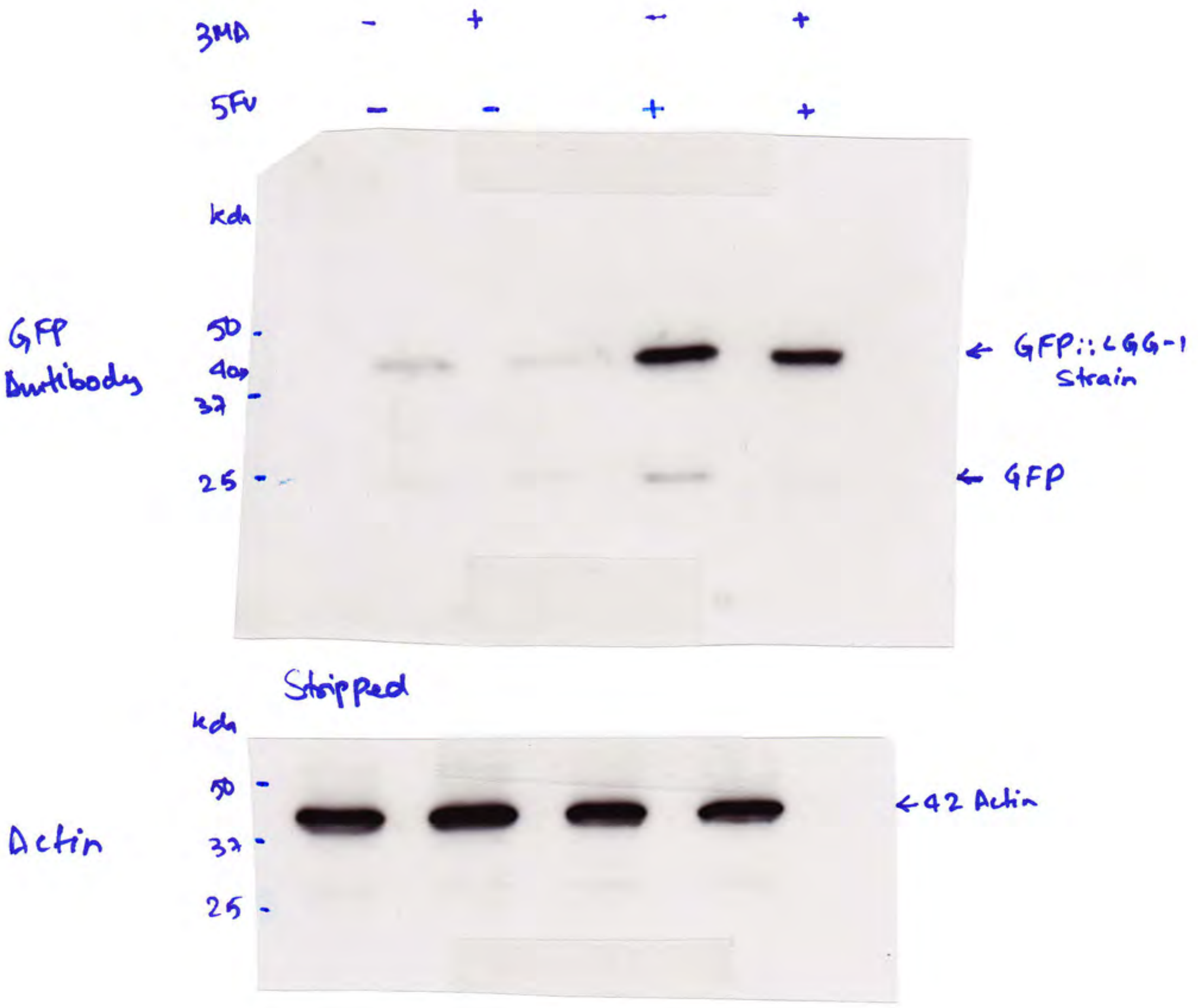

Original scans for Fig. 6 A.

Membrane cut in two:

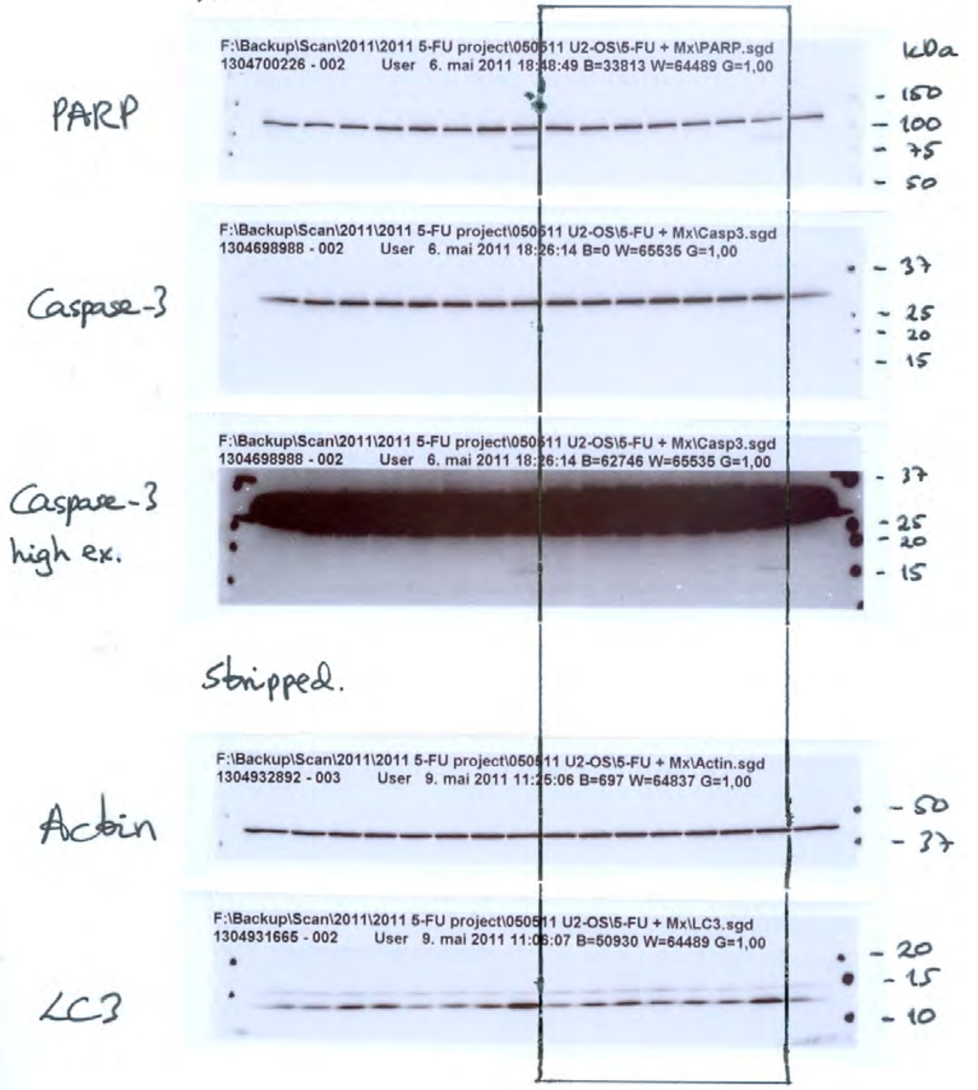

Original scans for Fig. 6E.

Membrane cut in three:

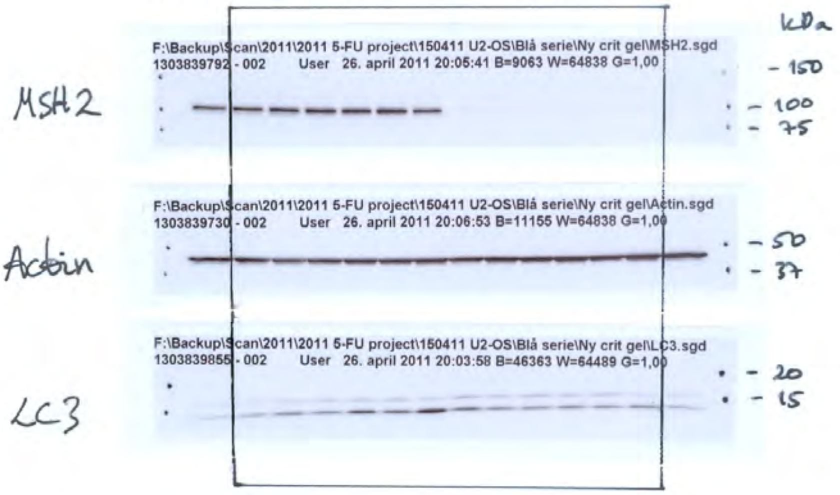

Original scans for supplementary Fig. S6A.

Membrane cut in three:

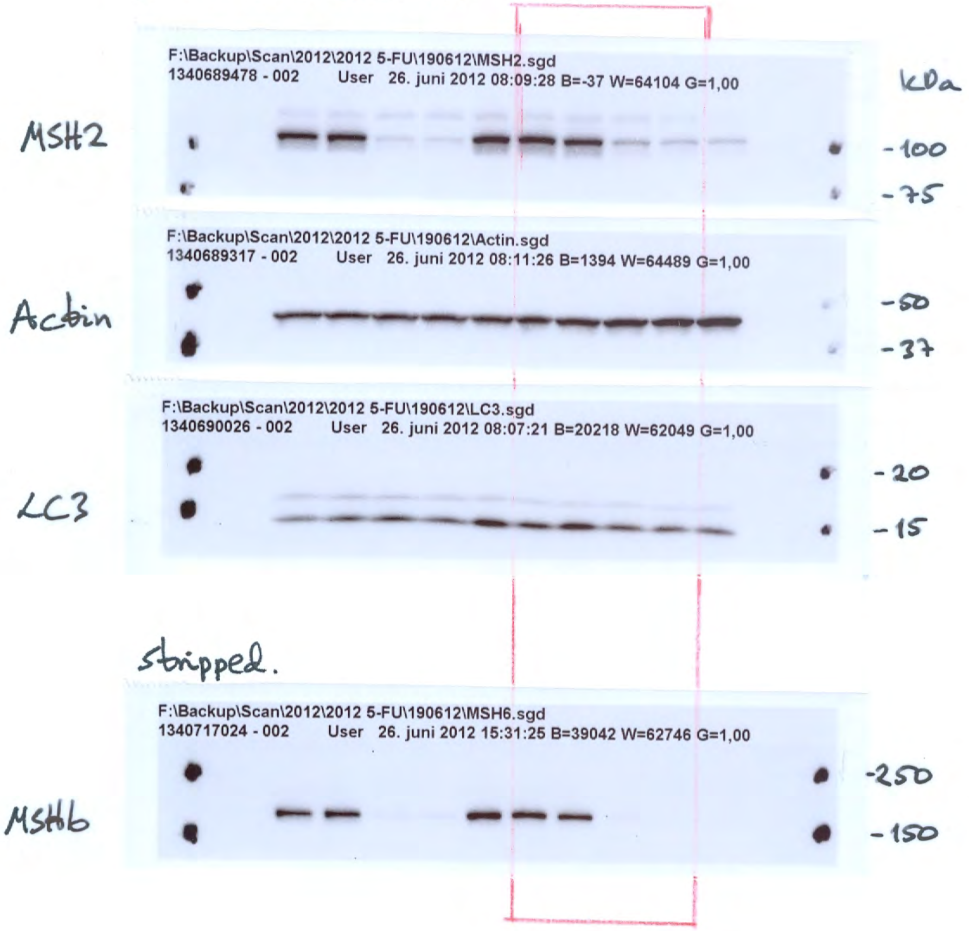

Original scans for supplementary Fig. S6 B.

Membrane cut in three, two pieces used:

RPS3

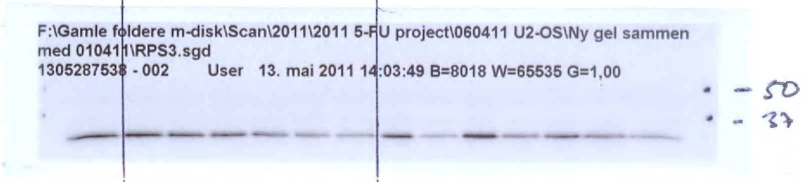

LC3

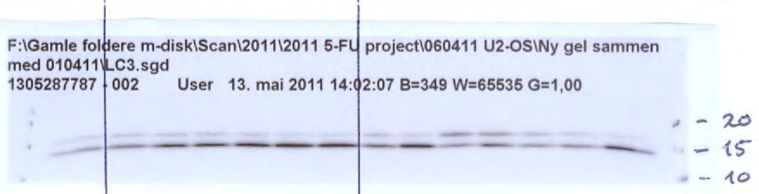

Stripped.

Actin

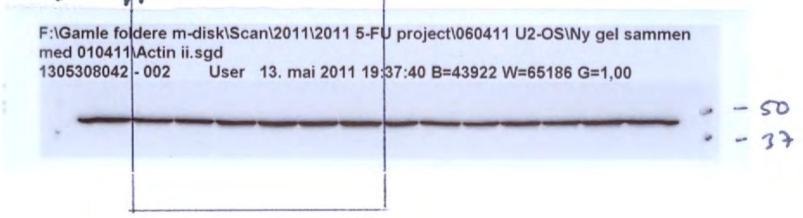

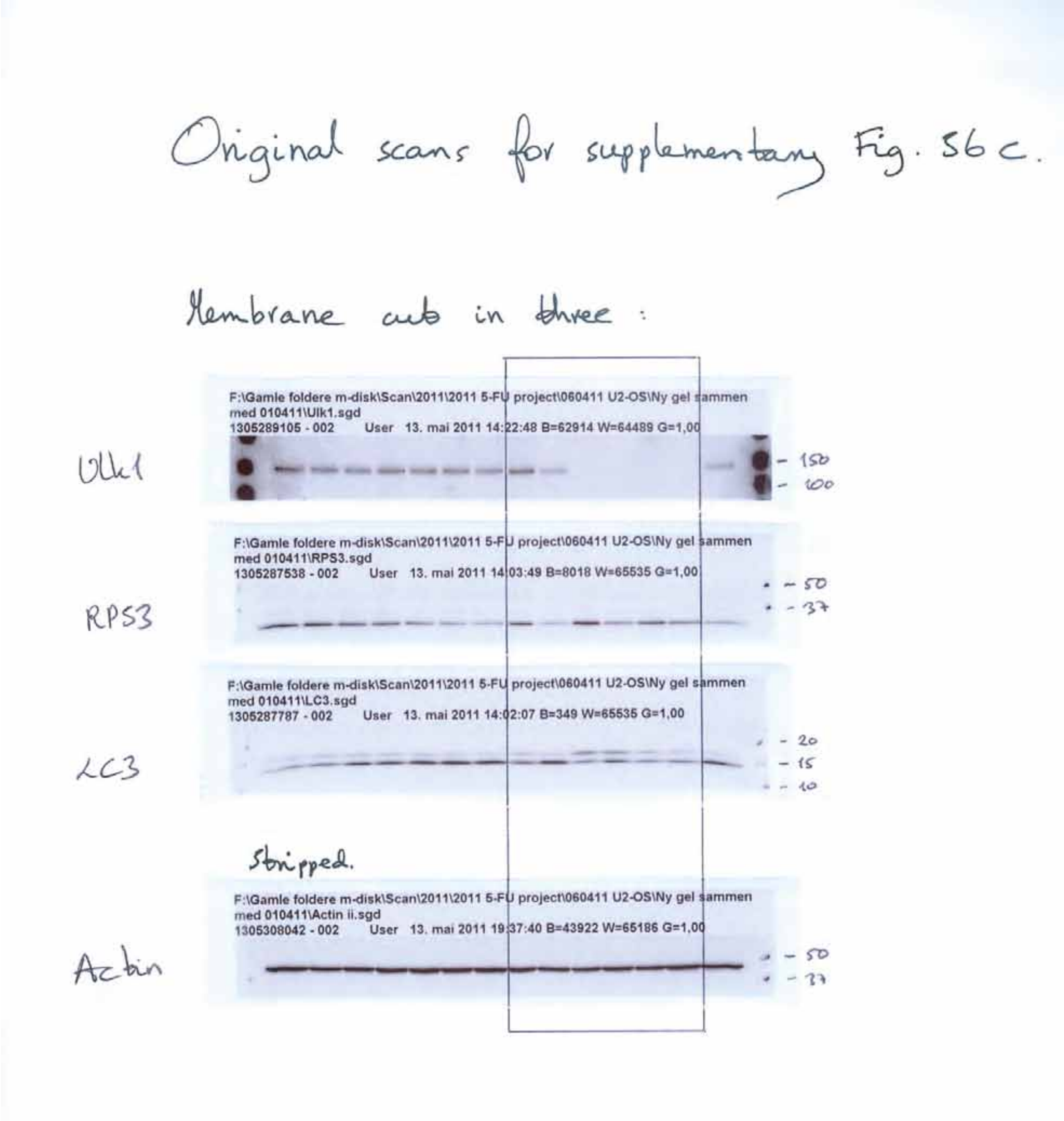

Supplementary Figure S8. Scanned images of all original western blots used in this study. The primary antibodies are written on the left of the panels.

Supplementary Table S1 *5-FU induced RPA-1 foci in embryos*

RNAi	*L4440*	*bec-1*	*ung-1*	*apn-1*	*exo-3*	*msh-6*	*atl-1*
No RPA foci	32	11	10	26	3	2	0
No embryos scored	35	13	10	35	14	17	11

Supplementary Table S2 *5-FU induced RPA-1 foci in embryos in mutants*

	Control RNAi			*apn-1(RNAi)*		
RNAi	N2	*mlh-1(ok1917)*	*exo-1(tm1842)*	N2	*mlh-1(ok1917)*	*exo-1(tm1842)*
No RPA-1+	32	0	1	26	0	0
No embryos scored	35	11	14	35	10	11

Supplementary Table S3 *Fraction of embryos with RAD-51 foci*

	4-6 cells stage			>100 cells stage		
RNAi	Untreated	IR	5-FU	Untreated	5-FU	IR
No RAD-51+	0	8	1	0	5	6
No embryos scored	6	13	7	0	7	6
RAD-51+ embryos (%)	0	63	14	0	71	100

Supplementary Table S4 *5-FU induced autophagy in embryos*

RNAi	*L4440*	*bec-1*	*unc-51*	*msh-2*	*msh-6*	*mlh-1*	*pms-2*	*ung-1*	*apn-1*	*exo-3*	*atl-1*	*cep-1*
No GFP+	245	19	13	74	13	10	22	141	31	48	75	70
No embryos scored	246	332	125	155	160	168	122	158	142	136	146	81
